# Synthesis and In Vitro Antiproliferative Activity of 11-Substituted Neocryptolepines with a Branched ω-Aminoalkylamino Chain

**DOI:** 10.3390/molecules22111954

**Published:** 2017-11-12

**Authors:** Elkhabiry Shaban, Marta Świtalska, Li Wang, Ning Wang, Fan Xiu, Ikuya Hayashi, Tran Anh Ngoc, Sachie Nagae, Samah El-Ghlban, Shiho Shimoda, Ahmed Abdel Aleem El Gokha, Ibrahim El Tantawy El Sayed, Joanna Wietrzyk, Tsutomu Inokuchi

**Affiliations:** 1 Division of Chemistry and Biotechnology, Graduate School of Natural Science and Technology, Okayama University, 3-1-1 Tsushima-naka, Kita-ku, Okayama 700-8530, Japan; shaban_nrc@yahoo.com (E.S.); liwang_512@163.com (L.W.); wn12171982@sina.com (N.W.); en20753@s.okayama-u.ac.jp (I.H.); trananhngoc90@gmail.com (T.A.N.); kunio.hearn@gmail.com (S.N.); S_elghlban@yahoo.com (S.E.-G.); n.n.n.ism.2@gmail.com (S.S.); 2 Hirszfeld Institute of Immunology and Experimental Therapy, Polish Academy of Sciences, 12, R. Weigla Street, 53-114 Wroclaw, Poland; switalska@iitd.pan.wroc.pl; 3 Department of Medicinal Chemistry, School of Pharmacy, Southwest Medical University, Luzhou 646000, China; 18090755675@163.com; 4 Department of Chemistry and Materials Engineering, Yingkou Institute of Technology, Yingkou 115014, China; 5 Department of Chemistry, Faculty of Science, Menoufia University, Shebin El Koom 32511, Egypt; aelgokha@yahoo.com

**Keywords:** indolo[2,3-*b*]quinolone, neocryptolepine, antiproliferative activity, SAR study, aminoalkylamino-substituted

## Abstract

Neocryptolepine, which is a kind of tetracyclic indoloquinoline alkaloid, exhibits the inhibition of topoisomerase II and shows antiproliferative activity. The present study describes the synthesis and antiproliferative evaluation of several neocryptolepine analogues carrying a branched, functionalized dibasic side chain at C11. These 2-substituted 5-methyl-indolo[2,3-*b*]quinoline derivatives were prepared by nucleophilic aromatic substitution (S_N_Ar) of 11-chloroneocryptolepines with appropriate 1,2- and 1,3-diamines. Some of the 11-(ω-aminoalkylamino) derivatives were further transformed into 11-ureido and thioureido analogues. Many of the prepared neocryptolepine derivatives showed submicromolar antiproliferative activity against the human leukemia MV4-11 cell line. Among them, 11-(3-amino-2-hydroxy)propylamino derivatives **2h** and **2k** were the most cytotoxic with a mean IC_50_ value of 0.042 μM and 0.057 μM against the MV4-11 cell line, 0.197 μM and 0.1988 μM against the A549 cell line, and 0.138 μM and 0.117 μM against the BALB/3T3 cell line, respectively.

## 1. Introduction

**Figure 1:**
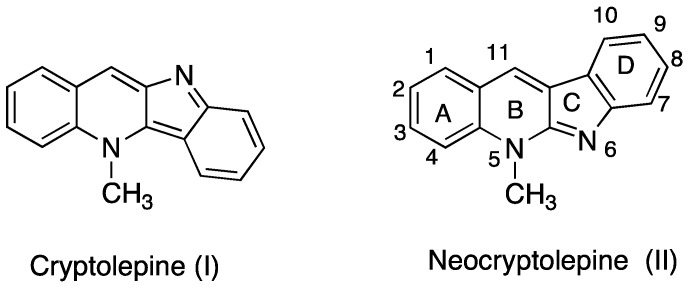
Chemical structures of indoloquinolines from Cryptolepis sanguinolenta.

The tetracyclic indoloquinoline ring systems constitute important structural motifs in natural products exhibiting numerous biological activities [1,2,3]. For example, cryptolepine (**I**, indolo[3,2-*b*]quinoline) and neocryptolepine (**II**, indolo[2,3-*b*]quinoline) are representative alkaloids isolated from the roots of the African plant *Cryptolepis sanguinolenta* [4,5,6] (Figure 1). Notably, an aqueous macerate or decoction of this plant is used in traditional medicine against malaria [7]. These two alkaloids, which only differ in the respective orientation of their indole and quinoline components, display potent antiparasitic properties [5,8,9,10]. Due to the linearly arranged planar tetracyclic structure, cryptolepine (**I**) and neocryptolepine (**II**) are DNA-intercalating agents and inhibit topoisomerase II, showing a high level of cytotoxicity [11,12,13,14].

Our previous structure activity relationship (SAR) study about the antiproliferative activity of the 5-methyl-indolo[2,3-*b*]quinoline derivatives revealed that the ω-aminoalkylamino substituent at C11 is an important element for their bioactivity. For example, the 3-aminopropylamino group on **2** (R^1^ = H) could increase the antiproliferative activity against the human leukemia cell line MV4-11 by about 20 times compared to that the of 11-chloro precursor **1** (R^1^ = H) [15,16].

Based on these findings, in this work, we further discuss the effect of the ω-aminoalkylamino substituent at C11 in the 5-methyl-indolo[2,3-*b*]quinolone core by diversifying the side-chain structure, i.e., changing the length and branching of the linker between the two nitrogen atoms, etc. We also examine whether a hydroxy group residing on the spacer exerts influence on the antiproliferative activity.

## 2. Results and Discussion

### 2.1. Synthesis

**Figure 2:**
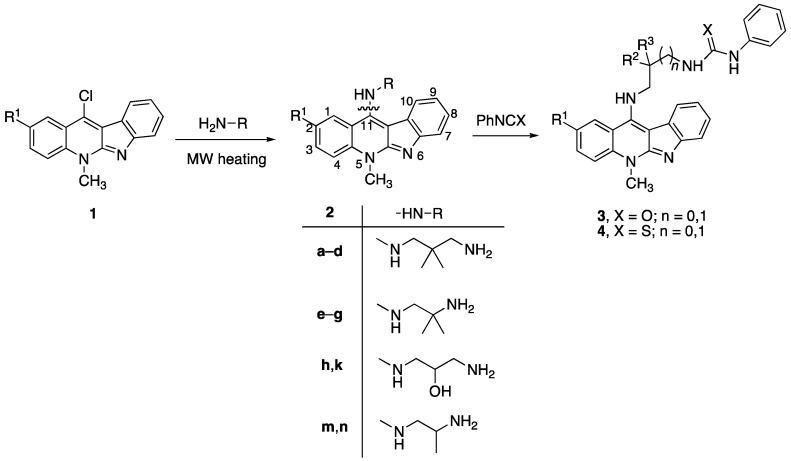
Synthesis of 11-aminoalkylamino-5-methyl-indolo[2,3-*b*]quinolines **2** and their ureido and thioureido derivatives **3** and **4**.

The synthetic strategy for the 5-methyl-indolo[2,3-*b*]quinoline derivatives **2** is based on the nucleophilic aromatic substitution (S_N_Ar) reaction of 11-chloroneocryptolepines **1** with an appropriate amine, as shown in Figure 2 [17]. The 11-chloroneocryptolepines **1**, the key intermediates for the diversification, were derived starting with various substituted *N*-methylanilines and methyl-1*H*-indole-3-carboxylates in three steps in good yields [18]. The amination of **1** with various 1,2-diaminoethanes and 1,3-diaminopropanes using an excess amount of dimethyl formamide (DMF), under heating, smoothly yielded the corresponding 11-aminated compounds **2**. Subsequently, a series of ureido derivatives **3** and thioureido derivatives **4** were prepared in high yields by modification of the free terminal amine group of **2** with phenylisocyanate and isothiocyanate in dry CH_2_Cl_2_ at room temperature, respectively [19].

### 2.2. Biological Evaluation

The natural product neocryptolepine (**II**) is known to exhibit prominent antiproliferative activities, for example, IC_50_ 12.7 ± 1.3 μM against the human (HL-60) leukaemia cell line [14] and IC_50_ 7.48 ± 4.42 μM against the human breast cancer MDA-MB-453 cell line [20], which are comparable with the reference anticancer drug cisplatin (IC_50_ 7.6 ± 0.7 μM). This antiproliferative activity is improved slightly by introduction of the Cl atom at the C11 of neocryptolepine core to IC_50_ 5.16 μM. Introduction of an amino group is expected to increase the cytotoxic activity compared to the non-substituted neocryptolepine. Accordingly, various kinds of amino groups were then evaluated to assess their antiproliferative activity.

The synthesized compounds **2a**–**2n** bearing various ω-aminoalkylamino groups at C11 were tested against the human leukemia MV4-11 cell line while varying substituents R^1^ at C2. As shown in Table 1, all assayed compounds were cytotoxic against MV4-11 leukemia cells (IC_50_ values below 0.9 μM), and exhibited higher antiproliferative activities than the reference anticancer drug cisplatin (IC_50_ 2.82 μM), but lower activities than doxorubicin HCl (IC_50_ 0.006 μM).

Amongst compounds **2a**–**2d** bearing a geminal dimethyl group in the pendant on the amino side chain of the three-carbon-atoms spacer, the Cl atom at C2 was favored over the methyl group (i.e., **2c** vs. **2b**) and the Br atom (i.e., **2c** vs. **2d**). However, amongst compounds **2e**–**2g** with a two-carbon-atoms spacer, compound **2f** bearing a methyl group at C2 was more effective than **2g** with a Cl atom at C2. Compound **2j**, bearing only one methyl group in the side chain on the two-carbon-atoms spacer, was more active than **2g** with a geminal methyl group in the side chain. Compounds **2m** and **2n** bearing a Cl or a Br atom at C2, respectively, showed almost the same activity. Amongst the tested compounds, **2h**, modified with a 3-amino-2-hydroxypropylamino group at C11 and a Cl at C2, showed the highest antiproliferative activity (IC_50_ 0.042 μM). 

Subsequently, the ureido and thioureido derivatives **3** and **4** were tested for their antiproliferative activity against the human leukemia MV4-11 cell line. Unfortunately, compounds **3** and **4** showed slightly less pronounced antiproliferative activities, compared with compound **2**, bearing a free terminal amino group (Table 2).

Compounds **2a**, **2c**, **2f**, **2h**, **2k**, **2m**, and **2n** being of high antiproliferative activity against MV4-11 cells were chosen as candidates for further studies on their antiproliferative activity against non-small cell lung cancer (A549) and colon cancer (HCT116) cell lines, along with normal murine fibroblasts (BALB/3T3). The test results are listed in Table 3. All tested compounds showed higher antiproliferative activities against the cancer cells than the cisplatin used as a control agent. Compounds **2k** and **2h** showed higher activity against the A549 and HCT116 cell line than other compounds, and they showed selective antiproliferative activity against the MV4-11 cell line. The 2-bromo-derivative **2n** and the 2-chloro-derivative **2m** showed almost the same cytotoxicity against A549 and HCT116 cell lines compared to the normal cell line BALB/3T3, but **2n** showed potent activity against the MV4-11 cell line with an IC_50_ value of 0.078 μM. Compounds **2c** and **2f** showed lower cytotoxicity against the normal cell line, and moderate activity against the A549 and HCT116 cell lines. It is quite obvious that the introduction of proper alkylamino substituents into biologically active derivatives can favourably influence their activities and selectivities in DNA binding.

### 2.3. Spectroscopic Characterization of Neocryptolepine Derivative ***2d*** Interacting with Salmon Sperm DNA

**Figure 3:**
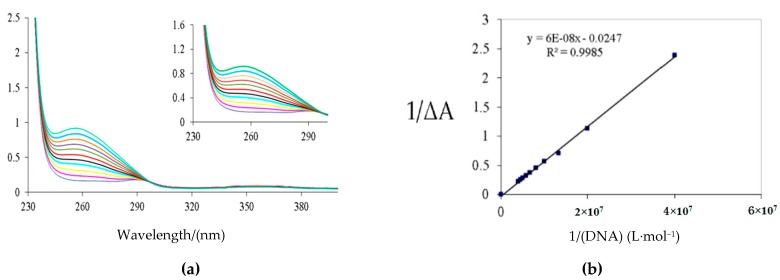
(**a**) UV-Vis absorption spectra of compound **2d** at 20 °C. C(**2d**) = 50 μmol/L, C(DNA) = 0.0, 0.025, 0.05, 0.075, 0.1, 0.125, 0.15, 0.175, 0.2, 0.225, 0.25 μmol/L for curves 1–11 in phosphate buffer solution (pH 7.0) (**b**) Plot of 1/ΔA vs. 1/[DNA] for **2d**-DNA.

The DNA binding studies of compound **2d** were performed using UV-Vis absorption spectroscopy with salmon sperm DNA in a phosphate buffer of pH 7.0 at 20 °C. A red shift and a hypochromic effect were observed in the absorption spectra while the DNA solution was gradually added to the solution of compound **2d**. The results depleted in Figure 3a showed that the minimum of the absorption band at 256 nm for **2d** decreased while increasing the DNA concentration. The maximum absorption shifted from 256 nm to 289 nm. This illustrated that the mode of **2d** binding to DNA was intercalation. The binding constant of **2d** to DNA was calculated as 4.12 × 10^5^ L·mol^−1^, according to a double-reciprocal equation. 

## 3. Experimental

### 3.1. Chemistry

The commercially obtained reagents (TCI, Tokyo, Japan) were used directly without further purification. The ^1^H-NMR and ^13^C-NMR spectra were taken on Varian INOVA-600, Varian INOVA-400, Varian INOVA-300 (Palo Alto, CA, USA), or Bruker 400 spectrometers (Billerica, MA, USA), using CDCl_3_ or DMSO-*d*_6_ as a solvent and tetramethylsilane (TMS) as the internal standard. The NMR spectra of the compounds are in Supplementary Materials. Mass spectra were obtained on a Bruker microTOF II-SKA spectrometer. The melting points were determined on a RFS-10 hot stage microscope (J-Science, Kyoto, Japan). The UV spectra were recorded with a U-2910 spectrophotometer (Hitachi, Tokyo, Japan) in a 1 cm path length quartz cuvette at an indicated wavelength, and the sample was dissolved in phosphate buffer with 2.5% DMSO. 

#### 3.1.1. General Procedure for the Synthesis of 11-Aminoneocryptolepines **2a**–**2n**

11-Chloroindoloquinolines **1** (0.3 mmol) and an excess of the appropriate ω-aminoalkylamine (3.0 mmol) were heated together at 120 °C for 4 h. Thin Layer Chromatography (TLC) monitoring was used to ensure the completion of the reaction. The resulting brown crude oil was purified by flash chromatography using AcOEt/2 M ammonia in MeOH (9:1, *v*/*v*) as an eluent to yield pure yellowish solid products.

*N-(3-Amino-2,2-dimethylpropyl)-5-methyl-5H-indolo[2,3-b]quinolin-11-amine* **2a**. Yellowish solid. Yield: 84%. M.p. 112–114 °C. ^1^H-NMR (400 MHz, CDCl_3_): δ = 8.18 (dd, *J* = 8.0, 4.0 Hz, 1H), 8.00 (d, *J* = 8.0 Hz, 1H), 7.76 (d, *J* = 8.0 Hz, 1H), 7.69–7.61 (m, 2H), 7.40 (t, *J* = 8.0 Hz, 1H), 7.32–7.28 (t, *J* = 8.0 Hz, 1H), 7.16 (t, *J* = 8.0 Hz, 1H), 4.23 (s, 3H), 3.85 (s, 2H), 2.86 (s, 2H), 0.90 (s, 6H). ^13^C-NMR (100 MHz, CDCl_3_): δ = 157.1, 152.2, 149.2, 138.1, 130.0, 125.0, 124.4, 121.7, 120.3, 118.5, 117.1, 116.1, 114.6, 105.0, 61.2, 53.0, 36.0, 32.7, 23.6 (2C). HRMS (ESI): *m*/*z* = 331.4462 [M − H]^−^, calcd. 332.4421.

*N-(3-Amino-2,2-dimethylpropyl)-2,5-dimethyl-5H-indolo[2,3-b]quinolin-11-amine* **2b**. Yellowish solid. Yield: 86 % M.p. 173–175 °C. ^1^H-NMR (400 MHz, CDCl_3_): δ = 8.01 (br s, 1H), 7.98 (d, *J* = 8.0 Hz, 1H), 7.74 (d, *J* = 8.0 Hz, 1H), 7.57–7.50 (m, 2H), 7.39 (t, *J* = 8.0 Hz, 1H), 7.14 (t, *J* = 8.0 Hz, 1H), 4.24 (s, 3H), 3.84 (s, 2H), 2.87 (s, 2H), 2.53 (s, 3H) 0.95 (s, 6H). ^13^C-NMR (100 MHz, CDCl_3_): δ = 149.0, 136.4, 131.4, 129.6, 125.1, 124.4, 123.9, 121.5, 118.3, 117.2, 116.0, 114.5, 106.0, 60.6, 52.8, 36.1, 32.6, 23.6 (2C), 21.1. HRMS (ESI): *m*/*z* = 347.2204 [M + H]^−^, calcd. 347.2236.

*N-(3-Amino-2,2-dimethylpropyl)-2-chloro-5-methyl-5H-indolo[2,3-b]quinolin-11-amine* **2c**. Yellowish solid. Yield: 95%. M.p. 177–179 °C. ^1^H-NMR (400 MHz, CDCl_3_): δ = 8.20 (s, 1H), 7.97(d, *J* = 8 Hz, 1H), 7.76 (d, *J* = 8 Hz, 1H), 7.60–7.56 (m, 2H), 7.41 (m, 1H), 7.18 (m, 1H), 4.22 (s, 3H), 3.85 (s, 2H), 2.92 (s, 2H), 0.90 (s, 6H). ^13^C-NMR (100 MHz, CDCl_3_): δ = 148.4, 136.8, 130.4 (2C), 126.3, 125.7 (2C), 124.7, 124.0 (2C), 122.4 (2C), 119.2, 117.5, 116.3, 62.0, 53.4, 36.0, 33.4, 24.0 (2C). HRMS (ESI): *m*/*z* = 365.1624 [M − H]^−^, calcd. 366.1611.

*N-(3-Amino-2,2-dimethylpropyl)-2-bromo-5-methyl-5H-indolo[2,3-b]quinolin-11-amine* **2d**. Yellowish solid. Yield: 86%. M.p. 184–186 °C. ^1^H-NMR (400 MHz, CDCl_3_): δ = 7.97 (q, *J* = 7.8 Hz, 2H), 7.76 (d, *J* = 7.8 Hz, 1H), 7.55 (q, *J* = 7.2 Hz, 1H), 7.40 (t, *J* = 7.5 Hz, 1H), 7.26 (t, *J* = 5.4 Hz, 1H), 7.19 (t, *J* = 7.8 Hz, 1H), 4.06 (s, 3H), 3.66 (s, 2H), 2.68 (s, 2H), 0.76 (s, 6H). ^13^C-NMR (75 MHz, CDCl_3_): δ = 157.1, 152.4, 148.0, 136.8, 132.7, 126.7, 125.4, 124.8, 122.5, 118.9, 117.7, 117.4, 116.3, 113.3, 104.6, 62.0, 53.2, 35.7, 33.1, 23.9 (2C).

*N-(2-Amino-2-methylpropyl)-5-methyl-5H-indolo[2,3-b]quinolin-11-amine* **2e**. Yellowish solid. Yield: 92%. M.p. 130–132 °C. ^1^H-NMR (400 MHz, CDCl_3_): δ = 8.24 (d, *J* = 8.0 Hz, 1H), 8.14 (d, *J* = 4.0 Hz, 1H), 7.80–7.71 (m, 3H), 7.43–7.41 (m, 1H), 7.33 (m, 1H), 7.01 (d, *J* = 8.0 Hz, 1H), 4.28 (s, 3H), 3.68 (S, 2H), 1.13 (s, 6H). ^13^C-NMR (100 MHz, CDCl_3_): δ = 156.8, 152.6, 149.5, 138.7, 130.6, 125.7, 125.1, 124.6, 121.5, 120.6, 119.2, 117.5, 115.8, 115.0, 106.9, 59.4, 51.6, 33.0, 29.4 (2C). HRMS (ESI): *m*/*z* = 319.1911 [M + H]^−^, calcd. 319.1923.

*N-(2-Amino-2-methylpropyl)-2,5-dimethyl-5H-indolo[2,3-b]quinolin-11-amine* **2f**. Yellowish solid. Yield: 88 %. M.p. 132–134 °C. ^1^H-NMR (400 MHz, CDCl_3_): δ = 8.13 (d, *J* = 7.6 Hz, 1H), 7.95 (s, 1H), 7.76 (d, *J* = 8.0 Hz, 1H), 7.53 (q, *J* = 8.0 Hz, 2H), 7.42 (t, *J* = 8.0 Hz, 1H), 7.19 (t, *J* = 7.6 Hz, 1H), 6.71 (s, 1H), 4.23 (s, 3H), 3.64 (d, *J* = 8.0 Hz, 2H), 2.55 (s, 3H), 1.10 (s, 6H). ^13^C-NMR (101 MHz, CDCl_3_): δ = 157.0, 153.0, 149.6, 137.0, 132.1, 130.2, 125.8, 124.7 (2C), 124.6, 121.6, 119.0, 117.5, 116.1, 115.1, 59.7, 51.9, 33.1, 29.6 (2C), 21.7. HRMS (ESI): *m*/*z* = 333.2054 [M + H]^−^, calcd. 333.2079.

*N-(2-Amino-2-methylpropyl)-2-chloro-5-methyl-5H-indolo[2,3-b]quinolin-11-amine* **2g**. Yellowish solid. Yield: 90%. M.p. 108–110 °C. ^1^H-NMR (400 MHz, CDCl_3_): δ = 8.15 (q, *J* = 7.6, 2H), 7.76 (d, *J* = 8.0 Hz, 1H), 7.62 (m, 2H), 7.44 (t, *J* = 7.6 Hz, 1H), 7.21 (t, *J* = 7.6 Hz, 1H), 6.73 (br, 1H), 4.22 (s, 3H), 3.58 (d, *J* = 4.5 Hz, 2H), 1.11 (s, 6H). ^13^C-NMR (101 MHz, CDCl_3_): δ = 156.4, 152.9, 148.2, 137.1, 130.4, 126.1, 125.9, 124.5, 124.4, 121.6, 119.4, 117.6, 116.8, 116.4, 107.6, 59.1, 51.5, 33.1, 29.5 (2C). HRMS (ESI): *m*/*z* = 351.1422 [M − H]^−^, calcd. 352.1455.

*1-Amino-3-(2-chloro-5-methyl-5H-indolo[2,3-b]quinolin-11-ylamino)propan-2-ol* **2h**. Yellowish solid. Yield: 82%. M.p. 150–152 °C. ^1^H-NMR (400 MHz, DMSO-*d*_6_): δ = 8.56 (s, 1H), 7.95 (d, *J* = 7.6 Hz, 1H), 7.85 (d, *J* = 9.2 Hz, 1H), 7.78 (q, *J* = 4 Hz, 1H), 7.50 (d, *J* = 8 Hz, 1H), 7.28 (t, *J* = 8 Hz, 1H), 7.07 (q, *J* = 8 Hz, 1H), 4.13 (s, 3H), 3.91–3.87 (m, 1H), 3.79 (dd, *J* = 12.8, 6.5 Hz, 2H), 3.69–3.66 (m, 2H). ^13^C-NMR (101 MHz, DMSO-*d*_6_): δ = 156.1, 152.4, 147.5, 136.1, 130.1, 124.9 (2C), 123.9, 123.4, 122.2, 118.1, 117.0, 116.7, 116.6, 105.4, 70.9, 52.3, 45.4, 32.3. HRMS (ESI): *m*/*z* = 354.1247 [M − H]^−^, calcd. 353.11275.

*1-Amino-3-(2-bromo-5-methyl-5H-indolo[2,3-b]quinolin-11-ylamino)propan-2-ol* **2k**. Yellowish solid. Yield: 76%. M.p. 162–164 °C. ^1^H-NMR (400 MHz, DMSO-*d*_6_): δ = 8.67 (s, 1H), 7.94 (t, *J* = 8.0 Hz, 2H), 7.81 (d, *J* = 8.0 Hz, 1H), 7.52 (d, *J* = 8.0, 1H), 7.30 (t, *J* = 7.8 Hz, 1H), 7.11 (t, *J* = 8.0 Hz, 1H), 4.13 (s, 3H), 3.87–3.82 (m, 1H), 3.81 (dd, *J* = 12.0, 6.5 Hz, 2H), 3.68–3.65 (m, 2H). ^13^C-NMR (101 MHz, DMSO-*d*_6_): δ = 156.1, 152.4, 147.5, 136.4, 132.8, 126.3 (2C), 124.9, 123.9, 122.2, 118.2, 117.3, 116.7, 112.7, 105.2, 70.9, 52.3, 45.4, 32.3.

*N-(2-Aminopropyl)-2-chloro-5-methyl-5H-indolo[2,3-b]quinolin-11-amine* **2m**. Yellowish solid. Yield: 87%. M.p. 95–79 °C. ^1^H-NMR (600 MHz, CDCl_3_): δ = 8.10 (d, *J* = 6 Hz, 2H), 7.76 (d, *J* = 7.8 Hz, 1H), 7.59 (t, *J* = 6.6 Hz, 1H), 7.54 (d, *J* = 9.1 Hz, 1H), 7.44 (t, *J* = 7.6 Hz, 1H), 7.22 (t, *J* = 7.2 Hz, 1H), 6.48 (br, 1H), 4.20 (s, 3H), 3.79 (d, *J* = 11.4 Hz, 1H), 3.40 (t, *J* = 10.8 Hz, 1H), 3.12 (q, *J* = 6 Hz, 1H), 1.10 (d, *J* = 6.2 Hz, 3H). ^13^C-NMR (151 MHz, CDCl_3_): δ = 156.3, 152.8, 147.9, 137.0, 130.4, 126.2, 125.9, 124.5, 124.3, 121.5, 119.4, 117.6, 116.9, 116.3, 108.1, 55.1, 47.9, 33.1, 22.9. HRMS (ESI): *m*/*z* = 337.1232 [M − H]^−^, calcd. 338.1298.

*N-(2-Amino-2-methylpropyl)-2-bromo-5-methyl-5H-indolo[2,3-b]quinolin-11-amine* **2n**. Yellowish solid. Yield: 90%. M.p. 101–103 °C. ^1^H-NMR (600 MHz, CDCl_3_): δ = 8.21 (s, 1H), 8.08 (d, *J* = 7.8 Hz, 1H), 7.74 (d, *J* = 7.8 Hz, 1H), 7.71 (q, *J* = 6 Hz, 1H), 7.48–7.43 (m, 2H), 7.20 (t, *J* = 7.8 Hz, 1H), 6.46 (s, 1H), 4.15 (s, 3H), 3.74 (d, *J* = 11.4 Hz, 1H), 3.37 (dd, *J* = 7.8, 7.8 Hz, 1H), 3.11–3.08 (m, 1H), 1.09 (d, *J* = 6.0 Hz, 3H). ^13^C-NMR (75 MHz, CDCl_3_): δ = 156.1, 152.6, 147.8, 137.3, 133.1, 127.5, 127.2, 126.2, 124.2, 121.5, 119.4, 117.6, 116.6, 113.3, 108.0, 55.1, 47.9, 33.2, 22.9.

#### 3.1.2. General Procedure for the Synthesis of **3a**–**3d** and **4a**,**b**

2-Substituted 5-methyl-5*H*-indolo]2,3-*b*]quinolone-11-amine (**2**) (50 mg) was dissolved in dry CH_2_Cl_2_ (1 mL), and a solution of phenylisocyanate or phenylisothiocyanate (1.1 equiv.) in dry CH_2_Cl_2_ (1 mL) was added drop by drop under stirring at room temperature for 2–4 h. TLC monitoring was used to ensure the completion of the reaction. The reaction mixture was evaporated to dryness under reduced pressure. The crude product was purified by flash chromatography using AcOEt/2M ammonia in MeOH (9:1, *v*/*v*) as an eluent to yield pure products as yellowish solids.

*1-(2,2-Dimethyl-3-(5-methyl-5H-indolo[2,3-b]quinolin-11-ylamino)propyl)-3-phenylurea* **3a**. Yellowish solid. Yield: 91%. M.p. 154–156 °C. ^1^H-NMR (600 MHz, CDCl_3_): δ = 9.25 (s, 1H), 8.42 (d, *J* = 6 Hz, 1H), 7.74–7.70 (m, 2H), 7.64 (d, *J* = 6 Hz, 1H), 7.46 (s, 2H), 7.40–7.35 (m, 2H), 7.24–7.22 (m, 2H), 7.14 (s, 1H), 6.97 (s, 1H), 6.77 (s, 1H), 6.64 (s, 1H), 4.03 (s, 3H), 3.60 (s, 2H), 3.25 (s, 2H), 2.29 (br, 1H), 0.63 (s, 6H). ^13^C-NMR (151 MHz, CDCl_3_): δ = 158.0, 157.1, 151.5, 149.6, 140.0, 137.9, 130.9, 129.5 (2C), 126.0, 124.4, 124.2, 123.1, 122.6, 121.9, 120.0 (2C), 119.7, 116.9, 116.5, 115.0, 106.8, 55.7, 48.0, 38.6, 33.3, 24.0 (2C). HRMS (ESI): *m*/*z* = 450.2364 [M − H]^−^, calcd. 451.2372.

*1-(3-(2,5-Dimethyl-5H-indolo[2,3-b]quinolin-11-ylamino)-2,2-dimethylpropyl)-3-phenylurea* **3b**. Yellowish solid. Yield: 83%. M.p. 228–230 °C. ^1^H-NMR (300 MHz, CDCl_3_): δ = 9.18 (s, 1H), 8.12 (s, 1H), 7.67 (d, *J* = 7.8 Hz, 1H), 7.57 (d, *J* = 7.8 Hz, 1H), 7.45 (d, *J* = 9 Hz, 3H), 7.34 (q, *J* = 6 Hz, 2H), 7.23 (d, *J* = 9 Hz, 2H), 7.08 (t, *J* = 9 Hz, 1H), 6.98 (t, *J* = 6 Hz, 1H), 6.49 (s, 1H), 6.10 (s, 1H), 3.97 (s, 3H), 3.52 (d, *J* = 6.6 Hz, 2H), 3.35 (d, *J* = 6.2 Hz, 2H), 2.58 (s, 3H), 0.77 (s, 6H). ^13^C-NMR (101 MHz, CDCl_3_): δ = 157.9, 156.4, 150.7, 149.7, 140.3, 136.0, 132.5, 131.6, 129.4 (2C), 125.9, 124.2 (2C), 124.0, 122.8, 122.6, 119.6 (2C), 116.8, 116.2, 114.9, 106.5, 55.7, 48.0, 38.6, 33.3, 24.1(2C), 21.7. HRMS (ESI): *m*/*z* = 466.4585 [M + H]^−^, calcd. 466.2607.

*1-(3-(2-Chloro-5-methyl-5H-indolo[2,3-b]quinolin-11-ylamino)-2,2-dimethylpropyl)-3-phenylurea* **3c**. Yellowish solid. Yield: 84%. M.p. 232–234 °C. ^1^H-NMR (400 MHz, CDCl_3_): δ = 8.36 (s, 1H), 8.13 (br, 1H), 7.73–7.70 (m, 2H), 7.60–7.57 (m, 1H), 7.45–7.39 (m, 3H), 7.39 (t, *J* = 7.4 Hz, 1H), 7.14–7.06 (m, 2H), 7.31 (t, *J* = 7.2 Hz, 2H), 6.53 (br, 1H), 5.91 (br, 1H), 4.11 (s, 3H), 3.57 (d, *J* = 6.4 Hz, 2H), 3.31 (d, *J* = 6.0 Hz, 2H), 0.73 (s, 6H). ^13^C-NMR (101 MHz, CDCl_3_): δ = 157.7, 156.8, 148.2, 139.7, 136.4, 130.9, 129.7 (2C), 127.3, 126.6, 124.0 (2C), 123.9, 123.7, 122.6, 120.6 (2C), 120.0, 117.9, 116.9, 116.4, 106.8, 55.9, 48.3, 38.4, 33.4, 24.1 (2C). HRMS (ESI): *m*/*z* = 484.1918 [M − H]^−^, calcd. 485.1982.

*1-(2-Methyl-1-(5-methyl-5H-indolo[2,3-b]quinolin-11-ylamino)propan-2-yl)-3-phenylurea* **3d**. Yellowish solid. Yield: 89%. M.p. 175–177 °C. ^1^H-NMR (600 MHz, CDCl_3_): δ = 8.88 (br, 1H), 8.31 (s, 1H), 7.73 (dd, *J* = 7.2, 7.2 Hz, 2H), 7.51 (t, *J* = 12 Hz, 4H), 7.37 (d, *J* = 8.4 Hz, 2H), 7.30 (t, *J* = 7.4 Hz, 2H), 7.10 (t, *J* = 7.2 Hz, 1H), 7.04 (t, *J* = 7.0 Hz, 1H), 6.05 (s, 1H), 4.07 (s, 3H), 3.77 (s, 2H), 1.17 (s, 6H). ^13^C-NMR (151 MHz, CDCl_3_): δ = 157.2, 156.9, 151.9, 148.7, 139.8, 136.5, 130.9, 129.6 (2C), 129.5, 127.1, 126.2, 124.3, 123.3, 122.3, 119.9 (2C), 119.7, 117.4, 117.0, 116.3, 106.4, 60.1, 54.6, 33.4, 26.3 (2C).

*1-(2,2-Dimethyl-3-(5-methyl-5H-indolo[2,3-b]quinolin-11-ylamino)propyl)-3phenylthiourea* **4a**. Yellowish solid. Yield: 90%. M.p. 172–174 °C. ^1^H-NMR (600 MHz, CDCl_3_): δ = 8.54 (d, *J* = 6 Hz, 1H), 7.86 (d, *J* = 6.0 Hz, 1H), 7.76 (dd, *J* = 7.8, 7.2 Hz, 2H), 7.66 (d, *J* = 7.8 Hz, 1H), 7.46 (t, *J* = 6.0 Hz, 1H), 7.39 (dd, *J* = 9, 7.2 Hz, 3H), 7.32 (d, *J* = 6.0 Hz, 2H), 7.25–7.19 (m, 1H), 6.89 (s, 1H), 4.24 (s, 3H), 3.79 (d, *J* = 6.0 Hz, 2H), 3.73 (d, *J* = 6.0 Hz, 2H), 1.96 (br, 1H), 0.71 (s, 6H). ^13^C-NMR (151 MHz, CDCl_3_): δ = 182.5, 153.4, 151.0, 145.5, 138.5, 137.4, 132.0, 129.5 (2C), 126.4, 126.24, 125.2 (2C), 125.0, 123.3, 123.1, 122.7, 121.1, 117.1, 115.4, 115.1, 103.5, 54.7, 51.8, 39.2, 34.6, 24.3 (2C). HRMS (ESI): *m*/*z* = 466.2170 [M − H]^−^, calcd. 467.2144.

*1-(1-(2-Chloro-5-methyl-5H-indolo[2,3-b]quinolin-11-ylamino)-2-methylpropan-2-yl)-3-phenylthiourea* **4b**. Yellowish solid. Yield: 84%. M.p. 147–149 °C. ^1^H-NMR (600 MHz, CDCl_3_): δ = 8.43 (s, 1H), 8.09 (s, 1H), 7.81 (d, *J* = 7.2 Hz, 1H), 7.68 (d, *J* = 8.4 Hz, 1H), 7.59–7.56 (m, 1H), 7.48 (d, *J* = 12 Hz, 1H), 7.38 (t, *J* = 7.6 Hz, 1H), 7.33–7.29 (m, 2H), 7.22 (t, *J* = 7.5 Hz, 1H), 7.11 (dd, *J* = 12.9, 7.5 Hz, 3H), 6.13 (s, 1H), 5.88 (s, 1H), 4.41 (d, *J* = 5.5 Hz, 2H), 4.06 (s, 3H), 1.22 (s, 6H). ^13^C-NMR (151 MHz, CDCl_3_): δ = 180.6, 156.5, 152.8, 147.9, 136.8 (d), 130.8, 130.4, 127.6, 126.8, 126.6, 125.5 (2C), 124.5, 124.4, 123.9, 121.7, 118.4, 117.6, 117.1, 116.6, 109.1, 60.8, 58.7, 55.5, 33.3, 26.7 (2C).

### 3.2. Antitumor Screening Test

Anti-proliferative assays in vitro were performed in the same manner as described in the literature [15,16,21,22]. The cell lines of human leukemia MV4-11, lung A549, and colon HCT 116 cancer were chosen as representing various tissue origins and BALB/3T3 normal murine fibroblasts to assess the cytotoxicity of tested compounds towards normal cells. The IC_50_ parameter (half-maximum inhibitory concentration) was calculated based on sulforhodamine B (HCT-116, A549, BALB/3T3) or MTT (MV4-11) assay results after 72 h of exposure [15,16]. Each compound in a given concentration was tested in triplicate in each experiment, which was repeated three to five times.

A lot of new chemicals undergo screening in various cell cultures but eventually do not achieve drug status due to severe side-effects. As Badisa et al. note: “Ideally, one of the criteria for a drug being good is that it should not exhibit any undesirable side-effects on normal cells” [23]. Therefore, to improve the search for new, not or significantly less toxic to normal mammalian cells compounds, the introduction of normal cells and a comparison of the cytotoxicity of tested compounds with cancer cells in early drug-screening protocols is valuable. Moreover, the BALB/3T3 fibroblasts used in our studies are recommended by various agencies introducing alternative methods for testing the toxicity of compounds. For example, these cells are used in some methods aiming to estimate starting doses for the oral acute systemic toxicity of compounds under the European Center of Validation of Alternative Methods (ECVAM) guidelines (OECD guidance document (GD) 129 published in 2010).

## 4. Conclusions

In conclusion, we have prepared a series of 11-substituted neocryptolepines with branched ω-aminoalkylamino chains of different linker lengths between the two nitrogen atoms, and their antiproliferative activities were evaluated using the MV4-11 (human leukemia), A549 (human lung cancer), HCT116 (human colon cancer), and normal mouse fibroblast (BALB/3T3) cell lines. All synthesized compounds showed potent in vitro antiproliferative activity against the MV4-11 cell line over the 11-chloro substituted precursors. As a result of the diversification of the ω-aminoalkylamino chains, we found the highest antiproliferative activity for 11-(3-amino-2-hydroxy)propylamino-substituted compounds **2h** and **2k,** showing mean IC_50_ values of 0.042 μM and 0.057 μM against MV4-11 cells, 0.197 μM and 0.1988 μM against A549 cells, and 0.138 μM and 0.117 μM against BALB/3T3 cells, respectively. The modification of ω-aminoalkylamino chains to ω-ureido and -thioureido derivatives was not the case for the improved activity. Further variations in substituents and their pattern may be necessary to obtain better activity.

## Figures and Tables

**Table 1:** Antiproliferative activity of 11-aminoalkylamino-5-methyl-indolo[2,3-*b*]quinolines against the human leukemia MV4-11 cell line.

Compound	R^1^	C11-Substituent	Yield, % of Amination	IC_50_ (μM)
Cisplatin		-		2.820 ± 0.450
Doxorubicin HCl		-	-	0.006 ± 0.002
**1a**	H	Cl	-	1.312 ± 0.262
**1b**	Br	Cl	-	0.810 ± 0.145
**2a**	H	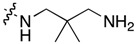	84	0.150 ± 0.060
**2b**	Me	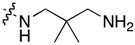	86	0.288 ± 0.075
**2c**	Cl	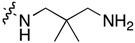	95	0.119 ± 0.043
**2d**	Br	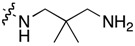	86	0.308 ± 0.102
**2e**	H	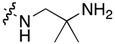	92	0.392 ± 0.188
**2f**	Me	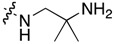	88	0.105 ± 0.027
**2g**	Cl	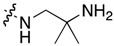	90	0.453 ± 0.209
**2h**	Cl	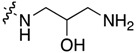	82	0.042 ± 0.014
**2k**	Br	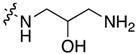	76	0.057± 0.015
**2m**	Cl	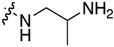	87	0.103 ± 0.014
**2n**	Br	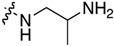	90	0.078 ± 0.020

The IC_50_ value is defined as the concentration of a compound that corresponds to a 50% growth inhibition. Data are expressed as the mean ± SD.

**Table 2:** Antiproliferative activity of the neocryptolepine derivatives **3a**–**3d**, **4a**, and **4b** against the human leukemia MV4-11 cell line.

Compound	n	R^1^	R^2^	R^3^	Yield, %	IC_50_ (μM)
**3a**	1	H	Me	Me	91 ^a^	0.549 ± 0.108
**3b**	1	Me	Me	Me	83 ^a^	0.427± 0.092
**3c**	1	Cl	Me	Me	84 ^a^	0.790 ± 0.302
**3d**	0	H	Me	Me	89 ^a^	0.464 ± 0.141
**4a**	1	H	Me	Me	90 ^b^	0.680 ± 0.215
**4b**	0	Cl	Me	Me	84 ^b^	2.330 ± 1.015

^a^ Yield of ureidation of the precursor amine **2**; ^b^ Yield of thioureidation of the precursor amine **2**. The IC_50_ value is defined as the concentration of a compound that corresponds to a 50% growth inhibition. Data are expressed as the mean ± SD.

**Table 3:** Antiproliferative activity of 11-alkylaminated 5-methyl-indolo[2,3-*b*]quinolines against normal mice fibroblasts BALB/3T3 and against the cancer cell lines A549 and HCT 116.

Compound	BALB/3T3 IC_50_ (μM)	A 549 IC_50_ (μM)	HCT 116 IC_50_ (μM)
Cisplatin	8.700 ± 0.097	9.870 ± 2.400	8.500 ± 0.540
Doxorubicin HCl	1.078 ± 0.033	0.329 ± 0.097	0.390 ± 0.098
**2a**	4.789 ± 2.018	1.512 ± 0.198	1.262 ± 0.361
**2c**	9.131 ± 0.844	1.455 ± 0.168	1.373 ± 0.351
**2f**	10.558 ± 0.330	1.795 ± 0.270	2.370 ± 0.481
**2h**	0.896 ± 0.042	0.197 ± 0.028	0.138 ± 0.050
**2k**	0.864 ± 0.015	0.190 ± 0.027	0.117 ± 0.055
**2m**	1.018 ± 0.017	1.269 ± 0.118	1.204 ± 0.283
**2n**	0.939 ± 0.018	0.988 ± 0.164	0.842 ± 0.367

The IC_50_ value is defined as the concentration of a compound that corresponds to a 50% growth inhibition. Human lung cancer cell line (A-549); human colon adenocarcinoma cell line (HCT 116); normal murine embryonic fibroblast cell line (BALB/3T3). Data are expressed as the mean ± SD.

## Supplementary Materials

The NMR spectra of the compounds can be available online.

## References

[1] Kumar E., Etukala J., Ablordeppey S. (2008). Indolo[3,2-*b*]quinolines: Synthesis, biological evaluation and structure activity relationships. Mini-Rev. Med. Chem..

[2] Lavrado J., Moreira R., Paulo A. (2010). Indoloquinolines as scaffolds for drug discovery. Curr. Med. Chem..

[3] Parvatkar P., Parameswaran S., Tilve S. (2011). Isolation, biological activities and synthesis of indoloquinoline alkaloids: Cryptolepine, isocryptolepine and neocryptolepine. Curr. Org. Chem..

[4] Cimanga K., Bruyne T., Pieters L., Claeys M., Vlietinck A. (1996). New alkaloids from *Cryptolepis sanguinolenta*. Tetrahedron Lett..

[5] Cimanga K., Bruyne T., Pieters L., Vlietinck A., Turger C. (1997). In Vitro and in vivo antiplasmodial activity of cryptoleipine and related alkaloids from *Cryptolepis sanguinolenta*. J. Nat. Prod..

[6] Paulo A., Gomes Elsa T., Steele J., Warhurst Dave C., Houghton Peter J. (2000). Antiplasmodial activity of *Cryptolepis sanguinolenta* alkaloids from leaves and roots. Planta Med..

[7] Anash C., Otsyina H., Duwiejue M., Woode E., Aboagye F., Aning K. (2009). Toxicological assessment of *Cryptolepis sanguinolenta* for possible use in veterinary medicine. J. Vet. Med. Anim. Health.

[8] Grellier P., Frappier F., Trigalo F., Ramiaramanana L., Millerioux V., Deharo E., Bodo B., Schrével J., Pousset J.L. (1996). Antimalarial activity of alkaloids isolated from *Cryptolepis sanguinolenta*, cryptolepine and isocryptolepine. Phytother. Res..

[9] Kirby G., Paine A., Warhurst D., Noamese B., Phillipson J. (1995). In vitro and in vivo antimalarial activity of cryptolepine, a plant-derived indoloquinoline. Phytother. Res..

[10] Wright C., Phillipson J., Awe S., Kirby G., Warhurst D. (1996). Antimalarial activity of cryptolepine and some other anhydronium bases. Phytother. Res..

[11] Guittat L., Alberti P., Rosu F., Van Miert S., Thetiot E., Pieters L., Gabelica V., De Pauw E., Ottaviani A., Riou J. (2003). Interactions of cryptolepine and neocryptolepine with unusual DNA structures. Biochimie.

[12] Jonckers T., Van Miert S., Cimanga K., Bailly C., Colson P., De Pauw-Gillet M., Van Den Heuvel H., Claeys M., Lemière F., Esmans E. (2002). Synthesis, cytotoxicity, and antiplasmodial and antitrypanosomal activity of new neocryptolepine derivatives. J. Med. Chem..

[13] Bailly C., Laine W., Baldeyrou B., De Pauw-Gillet M., Colson P., Houssier C., Cimanga K., Van Miert S., Vlietinck A., Pieters L. (2000). DNA intercalation, topoisomerase II inhibition and cytotoxic activity of the plant alkaloid neocryptolepine. Anti-Cancer Drug Des..

[14] Dassonneville L., Lansiaux A., Wattelet A., Wattez N., Mahieu C., Van Miert S., Pieters L., Bailly C. (2000). Cytotoxicity and cell cycle effects of the plant alkaloids cryptolepine and neocryptolepine: Relation to drug-induced apoptosis. Eur. J. Pharmacol..

[15] Wang L., Switalska M., Mei Z., Lu W., Takahara Y., Feng X., El-Sayed I., Wietrzyk J., Inokuchi T. (2012). Synthesis and in vitro antiproliferative activity of new 11-aminoalkylamino-substituted 5*H*- and 6*H*-indolo[2,3-*b*]quinolines; structure–activity relationships of neocryptolepines and 6-methyl congeners. Bioorg. Med. Chem..

[16] Lu W., Świtalska M., Wang L., Yonezawa M., El-Sayed I., Wietrzyk J., Inokuchi T. (2013). In vitro antiproliferative activity of 11-aminoalkylamino-substituted 5*H*-indolo[2,3-*b*]quinolines; improving activity of neocryptolepines by installation of ester substituent. Med. Chem. Res..

[17] El-Sayed I., Van der Veken P., Steert K., Dhooghe L., Hostyn S., Van Baelen G., Lemiere G., Maes B., Cos P., Maes L. (2009). Synthesis and antiplasmodial activity of aminoalkylamino-substituted neocryptolepine derivatives. J. Med. Chem..

[18] Wang L., Lu W., Odawara T., Misumi R., Mei Z., Peng W., El-Sayed I., Inokuchi T. (2014). Improved synthesis and reaction of 11-chloroneocryptolepines, strategic scaffold for antimalarial agent, and their 6-methyl congener from indolo-3-carboxylate. J. Heterocycl. Chem..

[19] Mei Z., Wang L., Lu W., Pang C., Maeda T., Peng W., Kaiser M., El-Sayed I., Inokuchi T. (2013). Synthesis and in vitro antimalarial testing of neocryptolepines: SAR study for improved activity by introduction and modification of side chains at C2 and C11 on 5H-indolo[2,3-*b*]quinolones. J. Med. Chem..

[20] Okada M., Mei Z.-W., Hossain M.I., Wang L., Tominaga T., Takebayashi T., Murakami M., Yasuda M., Shigehiro T., Kasai T. (2016). Synthesis, in-vitro cancer cell growth inhibition evaluation of 11-modified indolo[2,3-*b*]quinolines and their COMPARE analyses. Med. Chem. Res..

[21] Rubinstein L., Shoemaker P., Paull K., Simon M., Tosini S., Skehan P., Scudiero D., Monks A., Boyd M. (1990). Comparison of in vitro anticancer-drug–screening data generated with a tetrazolium assay versus a protein assay against a diverse panel of human tumor cell lines. J. Natl. Cancer Inst..

[22] Purcell M., Neault J., Tajmir-Riahi H. (2000). Interaction of taxol with human serum albumain. Biochim. Biophys. Acta Protein Struct. Mol. Enzymol..

[23] Badisa R., Darling-Reed S., Joseph P., Cooperwood J., Latinwo L., Goodman C. (2009). Selective cytotoxic activities of two novel synthetic drugs on human breast carcinoma MCF-7 cells. Anticancer Res..

