# Supplementary material for: Synthesis and In Vitro Antiproliferative Activity of 11-Substituted Neocryptolepines with a Branched ω-Aminoalkylamino Chain
Source: Molecules. 2017 Nov 12;22(11):1954. doi: 10.3390/molecules22111954 (PMC6150407; doi:10.3390/molecules22111954)
Supplement: Supplementary file 1 [file molecules-22-01954-s001.pdf]

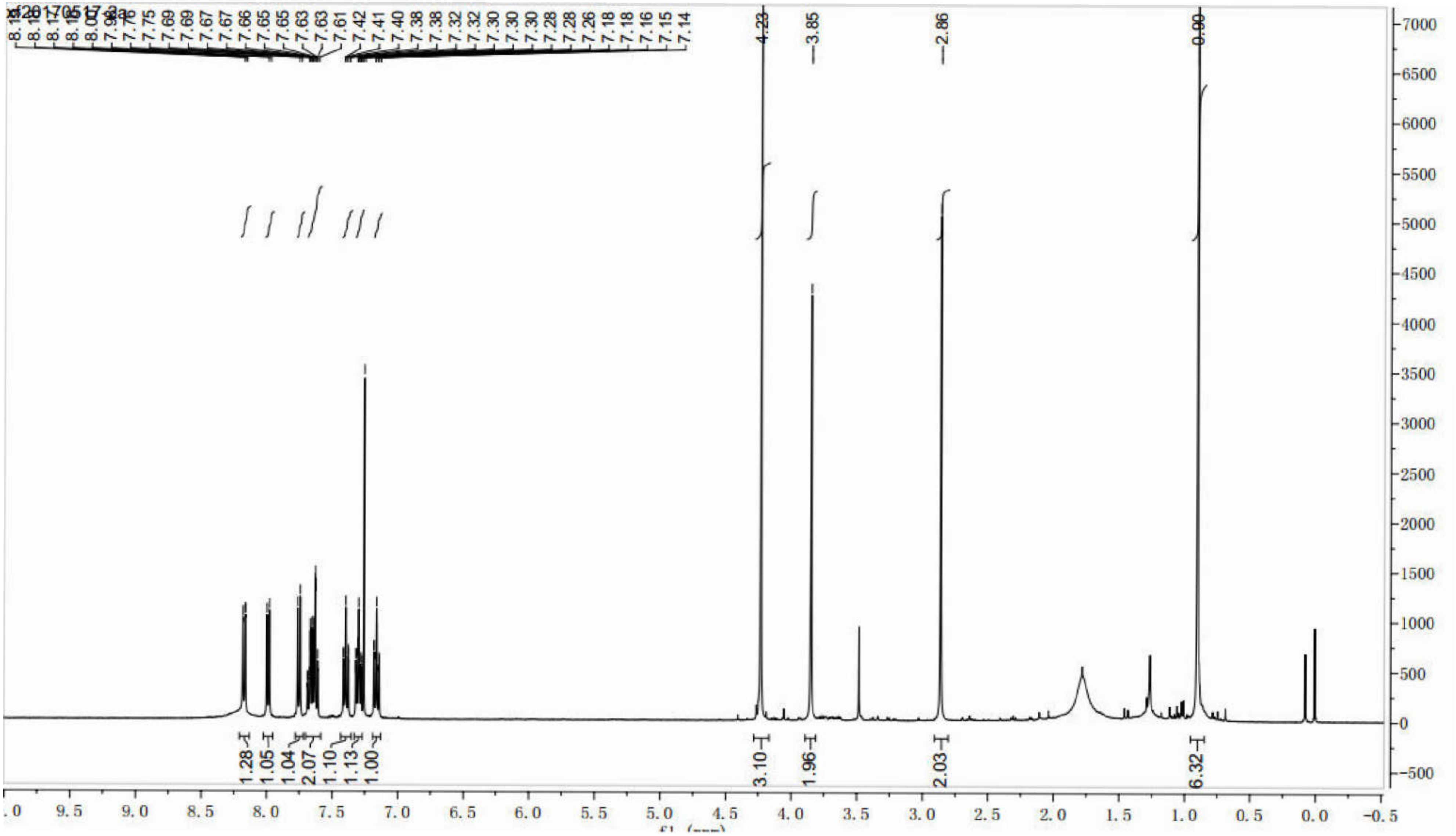

2a ^{1}H NMR

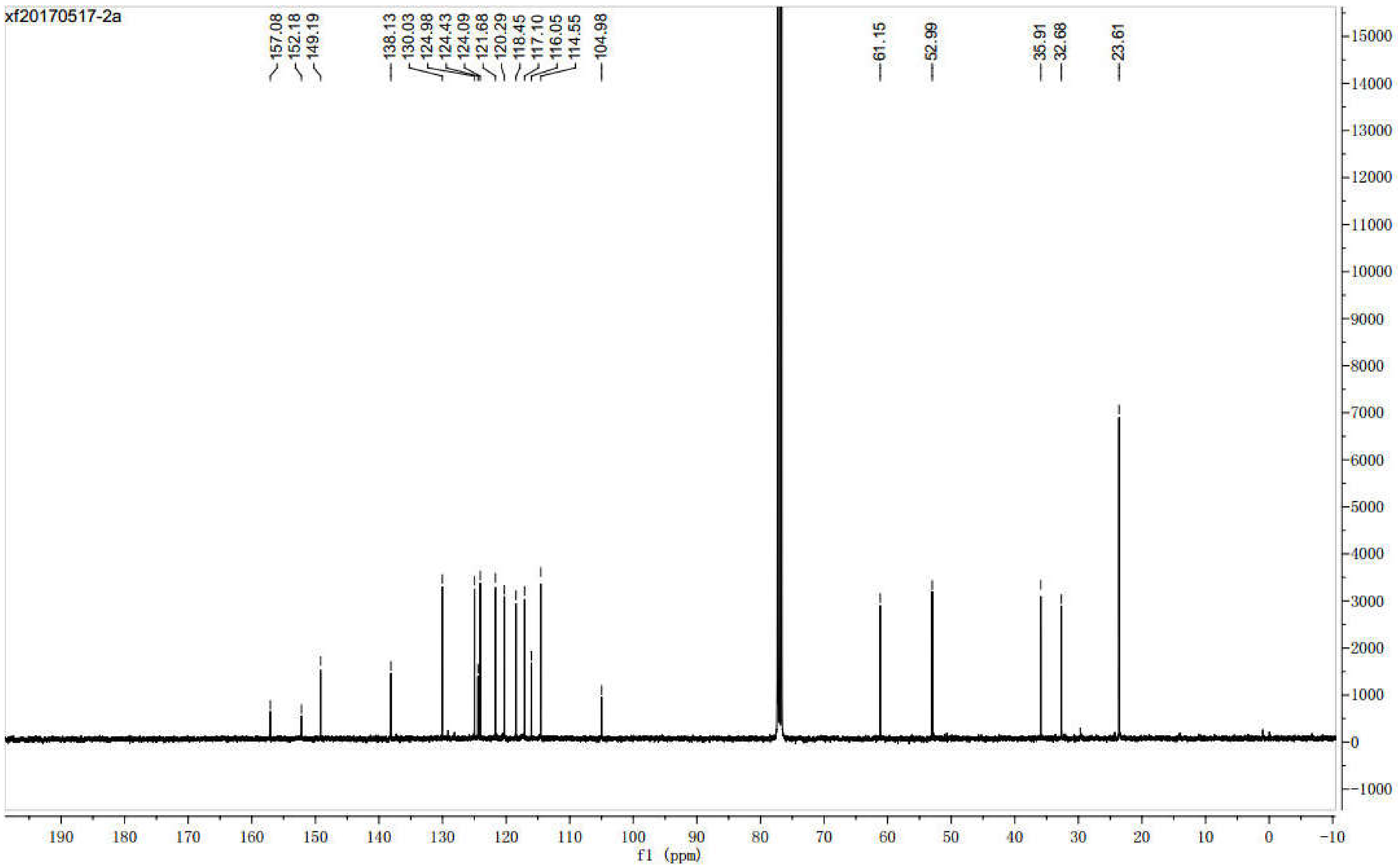
xf20170517-2a
157.08
152.18
149.19
138.13
130.03
124.98
124.43
124.09
121.68
120.29
118.45
117.10
116.05
114.55
104.98
61.15
52.99
35.91
32.68
23.61
f1 (ppm)

2a ^{13}C NMR

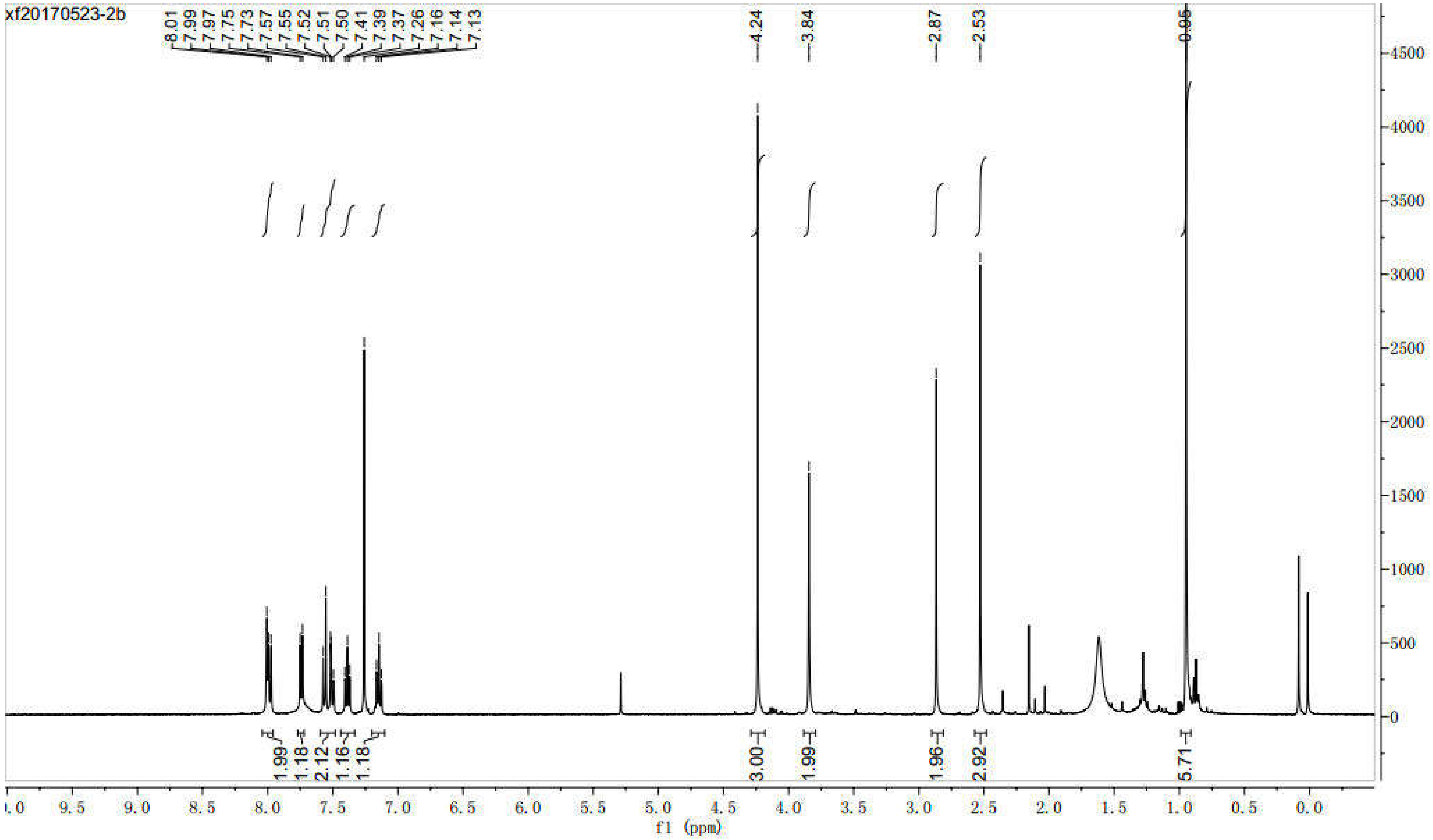
xf20170523-2b
8.01
7.99
7.97
7.75
7.73
7.57
7.55
7.52
7.51
7.50
7.41
7.39
7.37
7.26
7.16
7.14
7.13
4.24
3.84
2.87
2.53
0.96
1.99
1.18
2.12
1.16
1.18
3.00
1.99
1.96
2.92
5.71
f1 (ppm)

2b 1H NMR

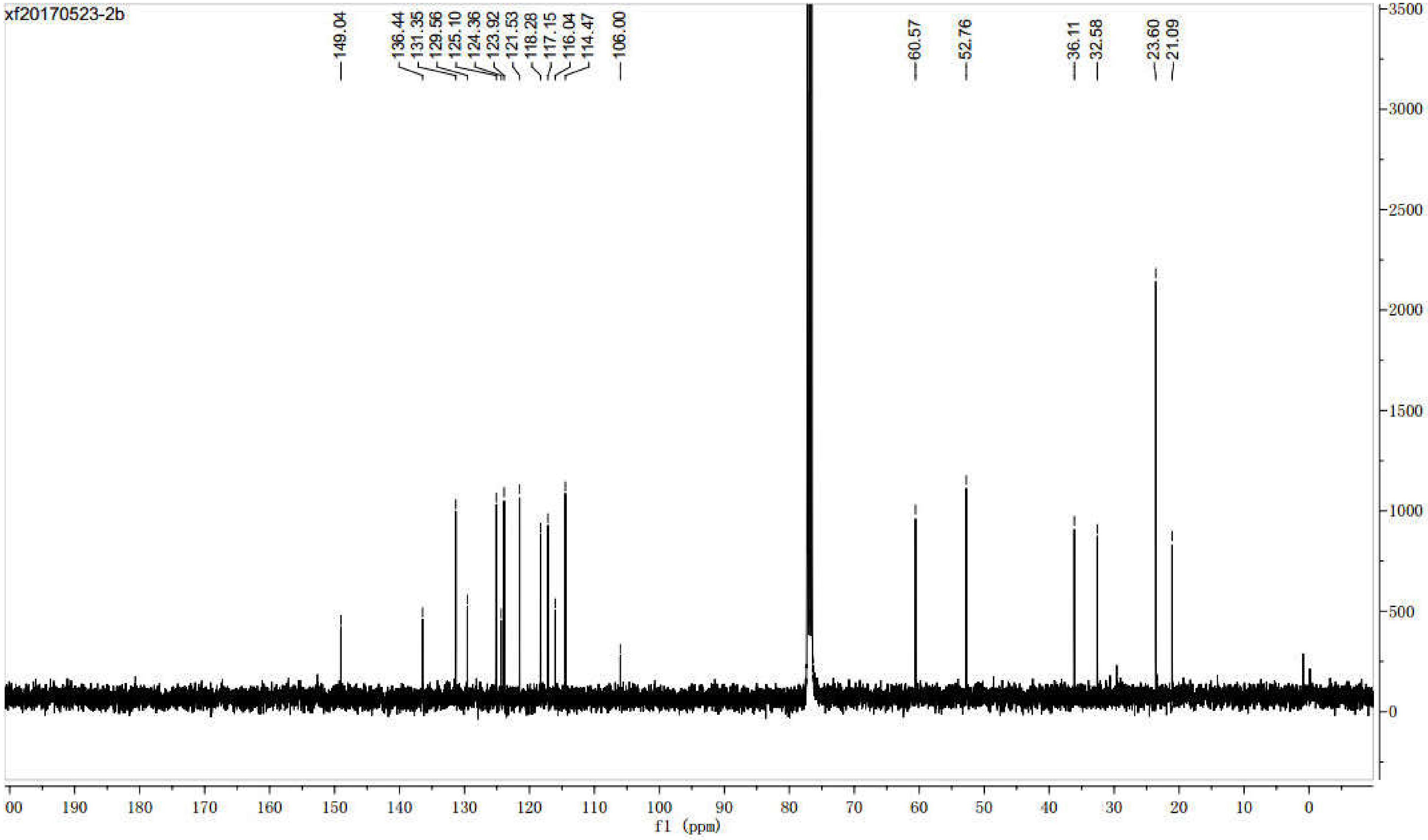
xf20170523-2b
149.04
136.44
131.35
129.56
125.10
124.36
123.92
121.53
118.28
117.15
116.04
114.47
106.00
60.57
52.76
36.11
32.58
23.60
21.09
3500
3000
2500
2000
1500
1000
500
0
00
190
180
170
160
150
140
130
120
110
100
90
80
70
60
50
40
30
20
10
0
f1 (ppm)

2b 13C NMR

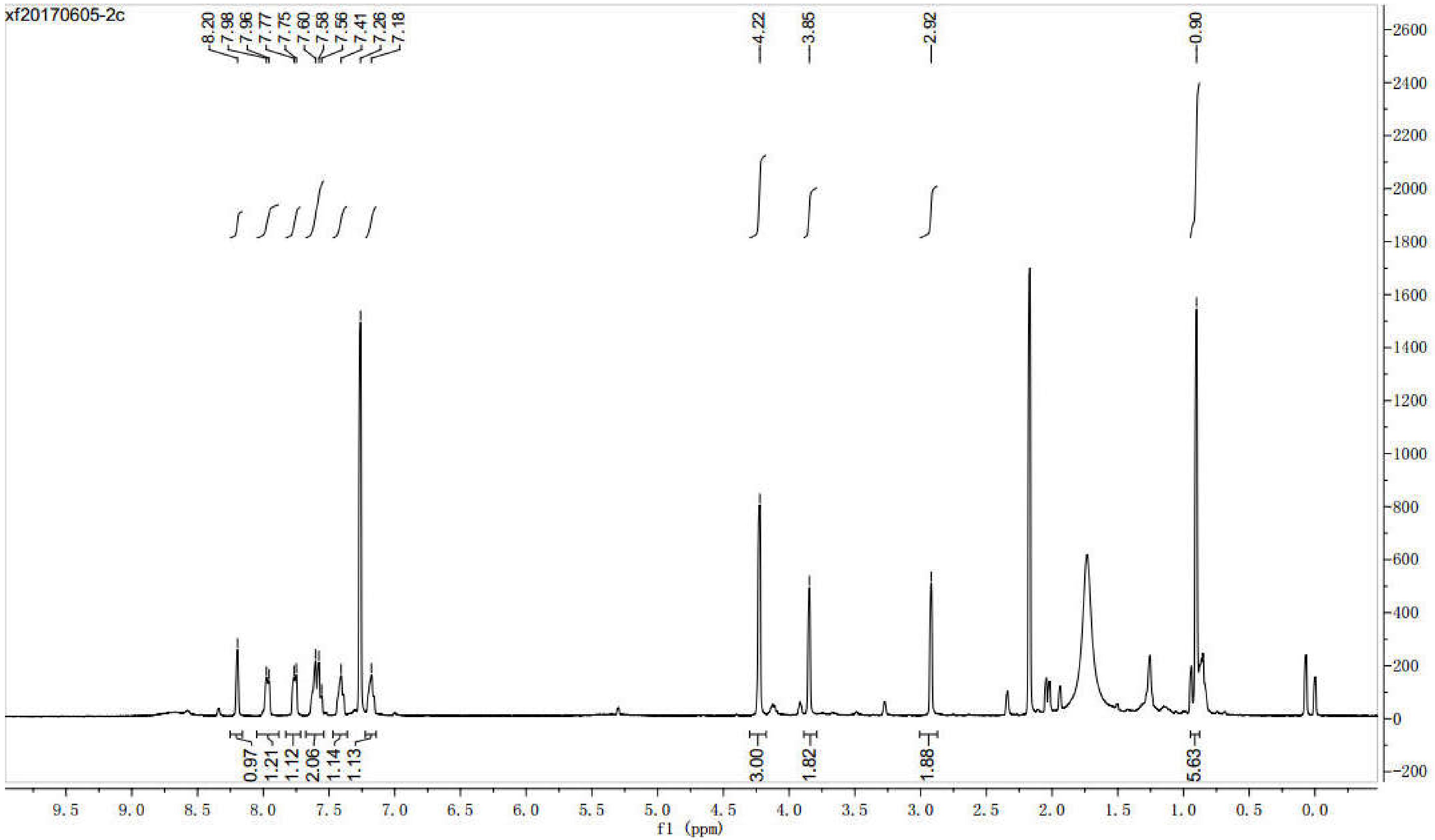
xf20170605-2c
8.20
7.98
7.96
7.77
7.75
7.60
7.58
7.56
7.41
7.26
7.18
4.22
3.85
2.92
0.90
0.97
1.21
1.12
2.06
1.14
1.13
3.00
1.82
1.88
5.63
f1 (ppm)

2c 1HNMR

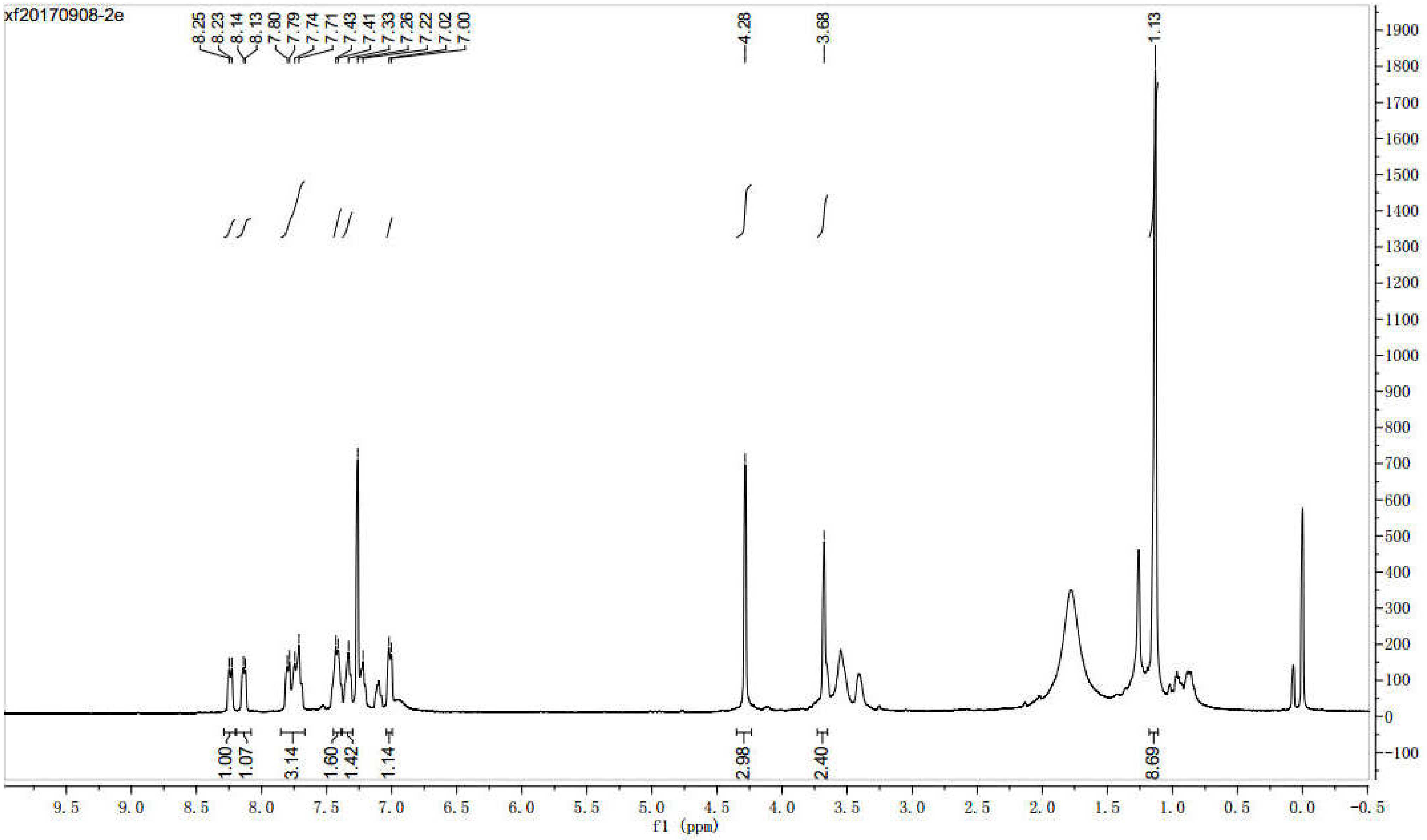
xf20170908-2e
8.25
8.23
8.14
8.13
7.80
7.79
7.74
7.71
7.43
7.41
7.33
7.26
7.22
7.02
7.00
4.28
3.68
1.13
1.00
1.07
3.14
1.60
1.42
1.14
2.98
2.40
8.69
f1 (ppm)

2e 1H NMR

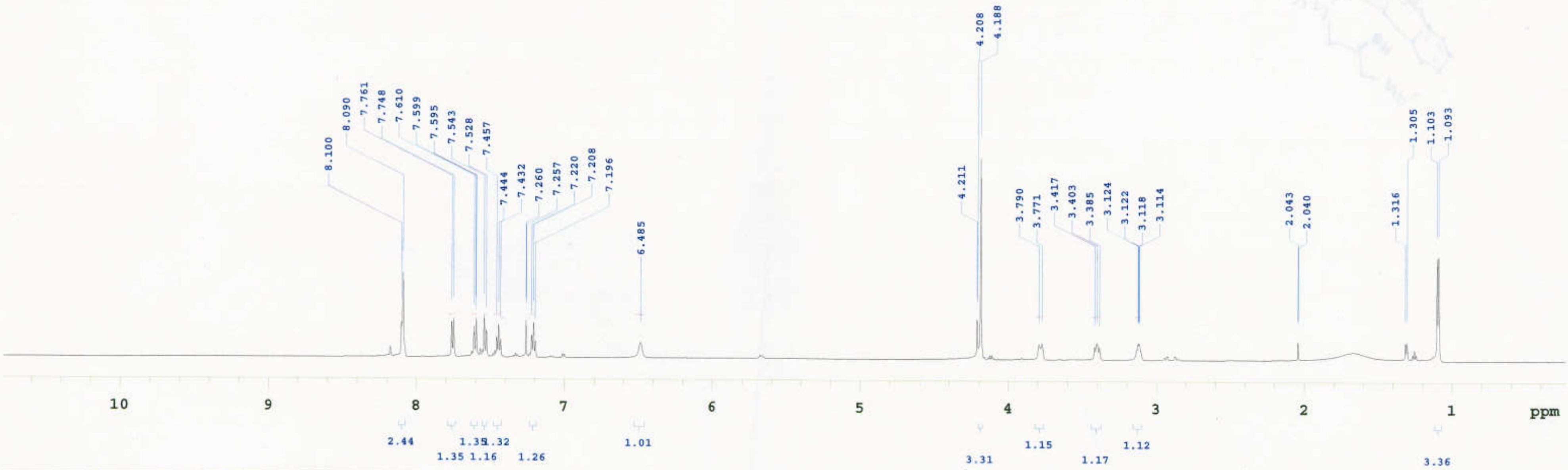

PULSE SEQUENCE
Relax. delay 1.592 sec
Pulse 45.0 degrees
Acq. time 3.408 sec
Width 9615.4 Hz
16 repetitions

OBSERVE H1, 599.7712267

DATA PROCESSING
Line broadening 0.3 Hz
FT size 65536
Total time 1 minute

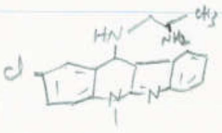

2 m

Solvent: cdcl3
Ambient temperature
Operator: walkup
VNMRS-600 "varian600"

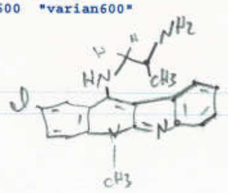

sh 585

File: exp

Pulse Sequence: s2pul

File: Data not saved yet

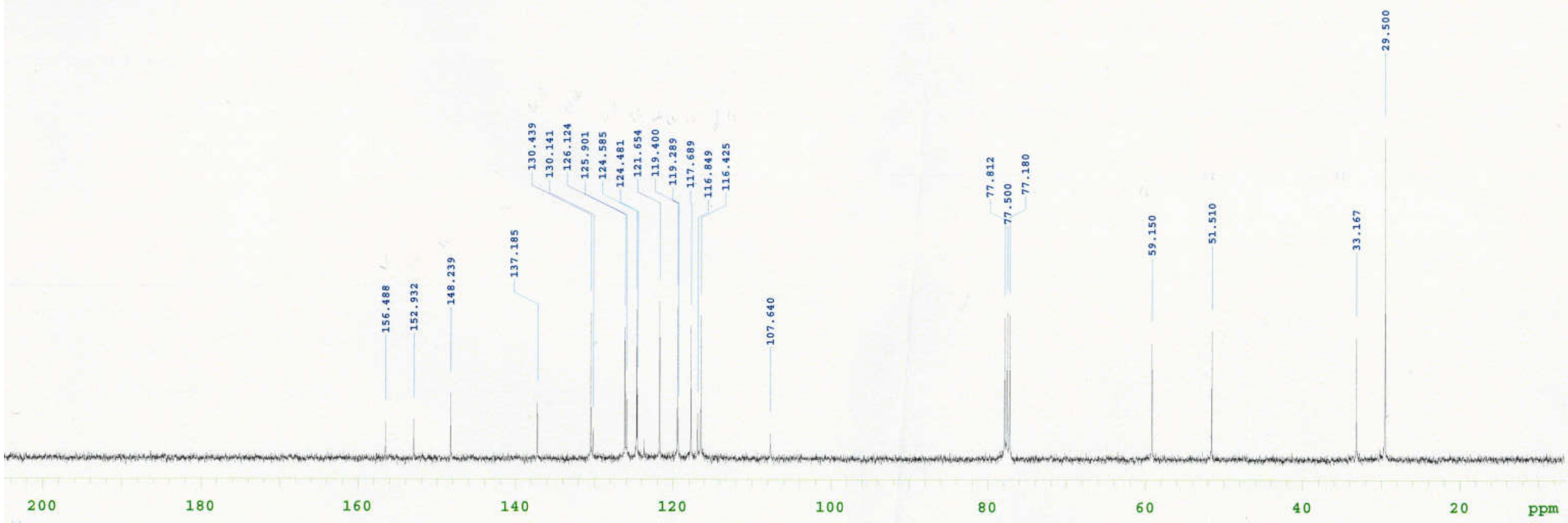

)0.00

ULSE SEQUENCE
Relax. delay 0.700 sec
Pulse 45.0 degrees
Acq. time 1.300 sec
Width 24509.8 Hz
512 repetitions

OBSERVE C13, 100.5573036
DECOUPLE H1, 399.9115921
Power 42 dB
continuously on
WALTZ-16 modulated

DATA PROCESSING
Line broadening 1.0 Hz
FT size 65536
Total time 17 minutes

2g

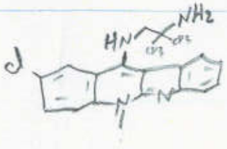

Solvent: cdcl3
Ambient temperature
Operator: walkup
VNMRS-400 "Varian-400MR"

sh572 c13
File: exp
Pulse Sequence: s2pul

File: /home/walkup/vnmrsys/data/data/s2pul_001.fid

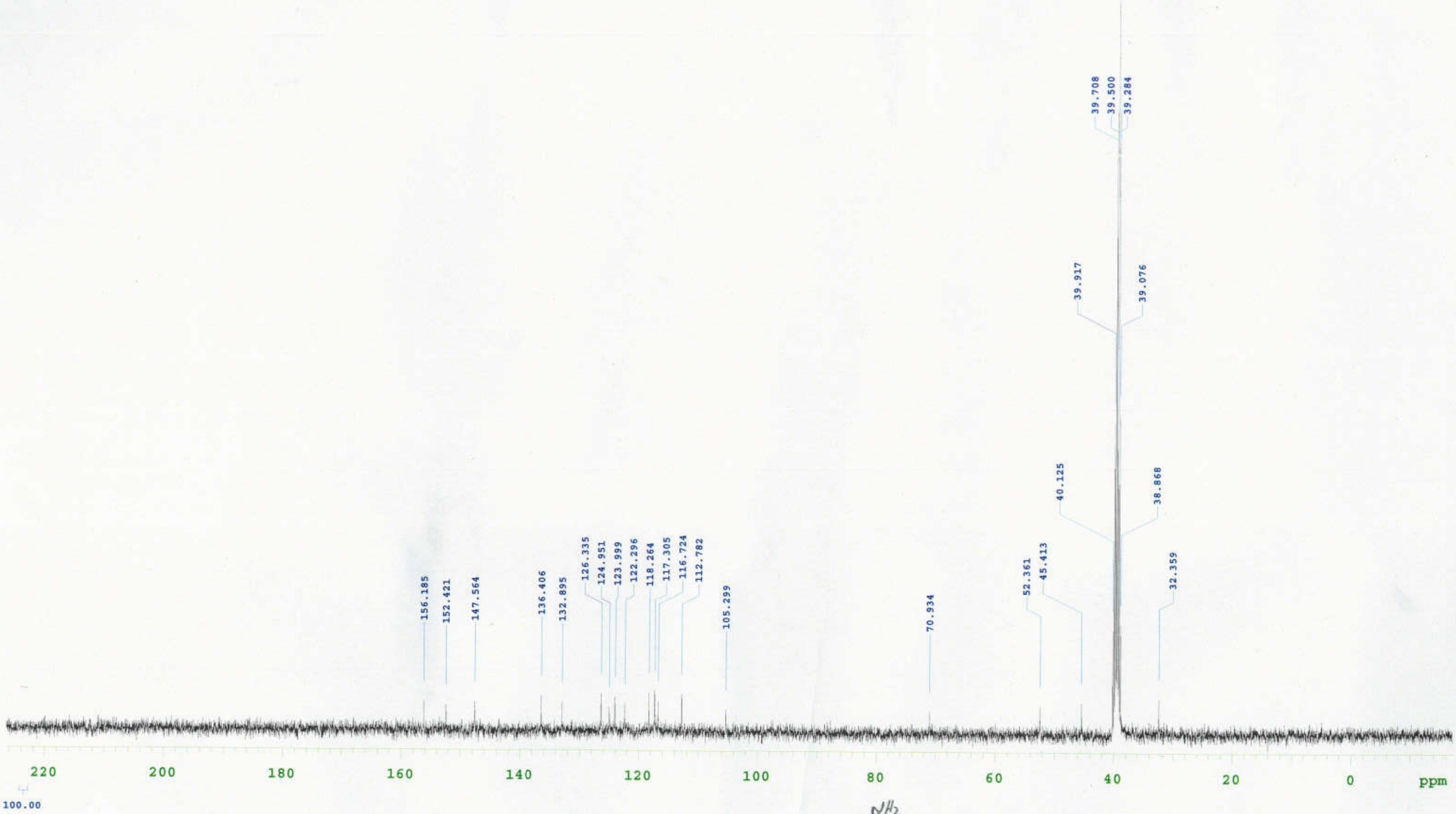

PULSE SEQUENCE
Relax. delay 0.700 sec
Pulse 45.0 degrees
Acq. time 1.300 sec
Width 24509.8 Hz
512 repetitions

OBSERVE C13, 100.5578678
DECOUPLE H1, 399.9134917
Power 42 dB
continuously on
WALTZ-16 modulated

DATA PROCESSING
Line broadening 1.0 Hz
FT size 65536
Total time 17 minutes

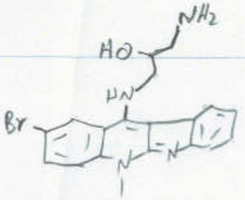

Solvent: dmso
Ambient temperature
Operator: vnmr1
VNMRS-400 "Varian-400MR"

sh 590

File: xp

Pulse Sequence: s2pul

File: Data not saved yet

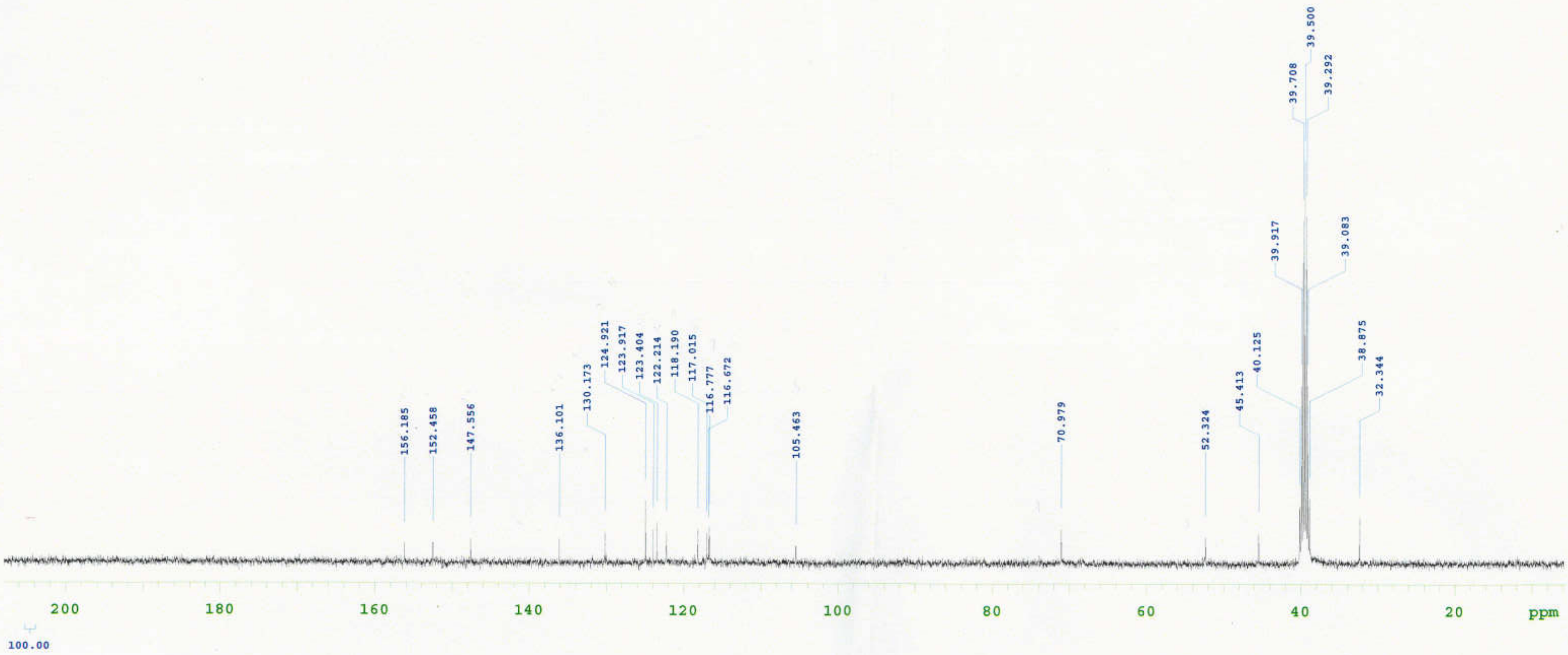

PULSE SEQUENCE
Relax. delay 0.700 sec
Pulse 45.0 degrees
Acq. time 1.300 sec
Width 24509.8 Hz
512 repetitions

OBSERVE C13, 100.5578731
DECOUPLE H1, 399.9134917
Power 42 dB
continuously on
WALTZ-16 modulated

DATA PROCESSING
Line broadening 1.0 Hz
FT size 65536
Total time 17 minutes

2h

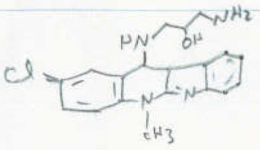

Solvent: dmso
Ambient temperature
Operator: walkup
VNMRS-400 "Varian-400MR"

sh575 c13

File: exp

Pulse Sequence: s2pul

File: /home/walkup/vnmrsys/data/data/s2pul_001.fid

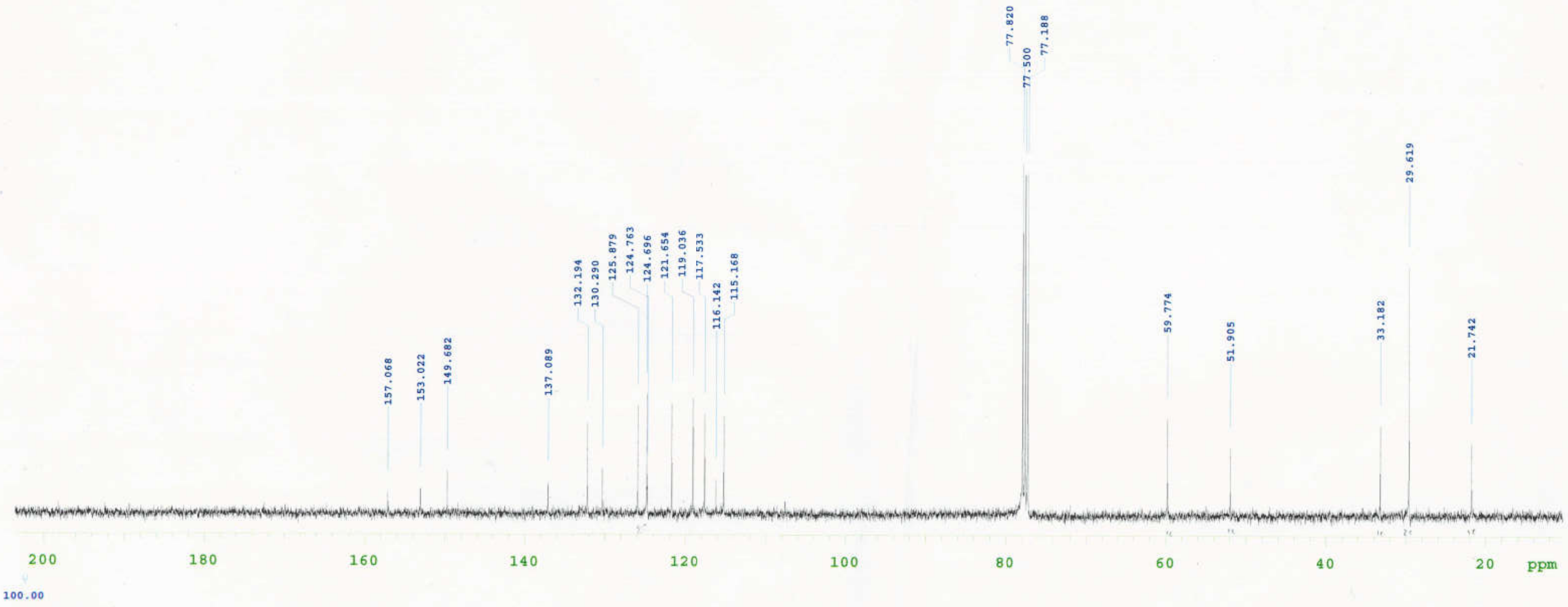

PULSE SEQUENCE	OBSERVE C13, 100.5572969	DATA PROCESSING		Solvent: cdcl3	
Relax. delay 0.700 sec	DECOUPLE H1, 399.9115921	Line broadening 1.0 Hz		Ambient temperature	
Pulse 45.0 degrees	Power 42 dB	FT size 65536		Operator: walkup	sh 569 c 13
Acq. time 1.300 sec	continuously on	Total time 33 minutes		VNMRS-400 "Varian-400MR"	File: exp
Width 24509.8 Hz	WALTZ-16 modulated	2F			Pulse Sequence: s2pul
1000 repetitions					

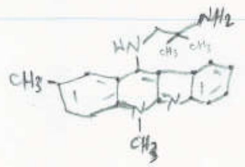

File: /home/walkup/vnmrsys/data/data/s2pul_001.fid

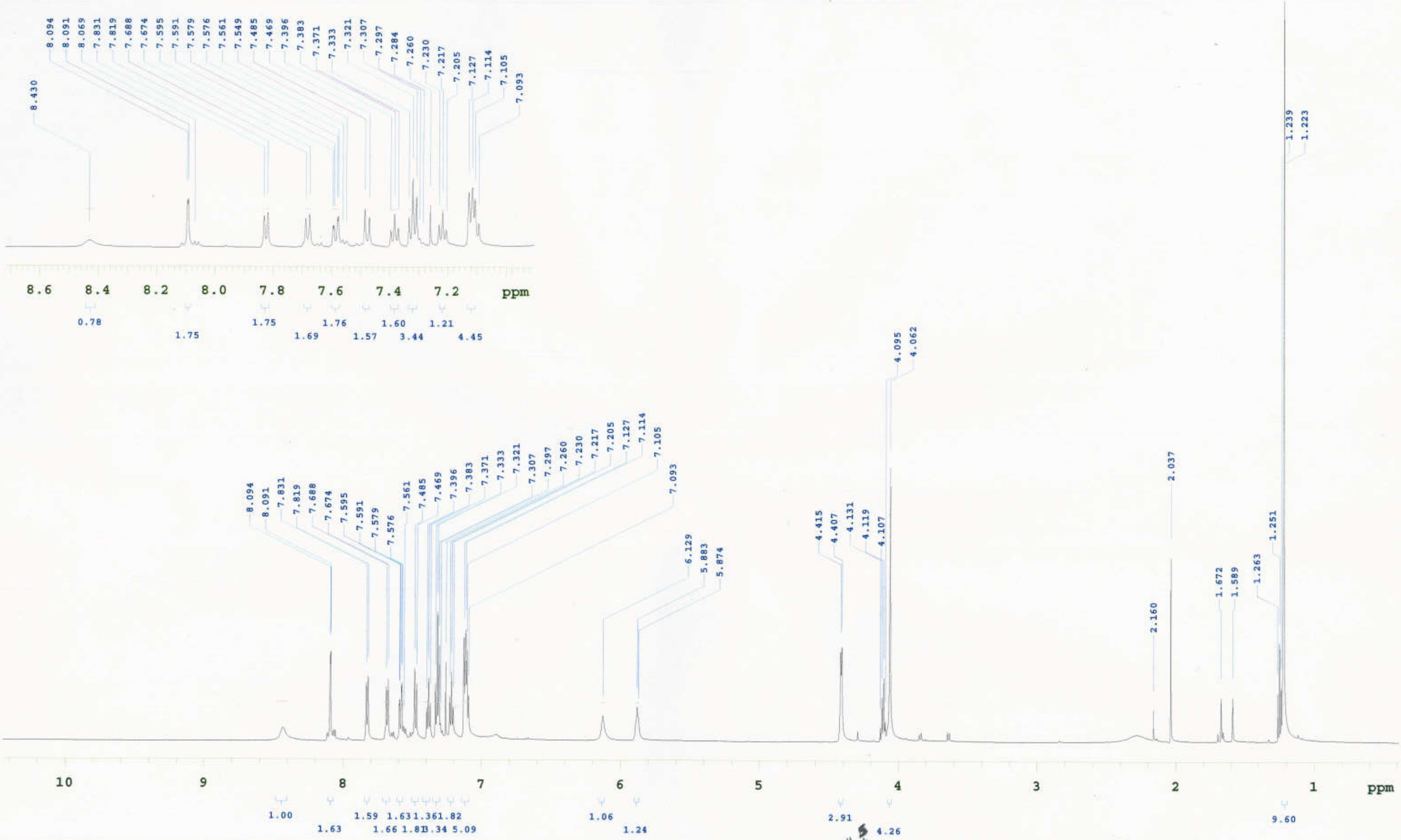

PULSE SEQUENCE
Relax. delay 1.592 sec
Pulse 45.0 degrees
Acq. time 3.408 sec
Width 9615.4 Hz
16 repetitions

OBSERVE H1, 599.7712258

DATA PROCESSING
Line broadening 0.3 Hz
FT size 65536
Total time 1 minute

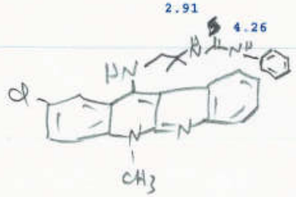

Solvent: cdcl3
Ambient temperature
Operator: walkup
VNMRS-600 "varian600"

sh 574

File: Data not saved yet

Pulse Sequence: s2pul

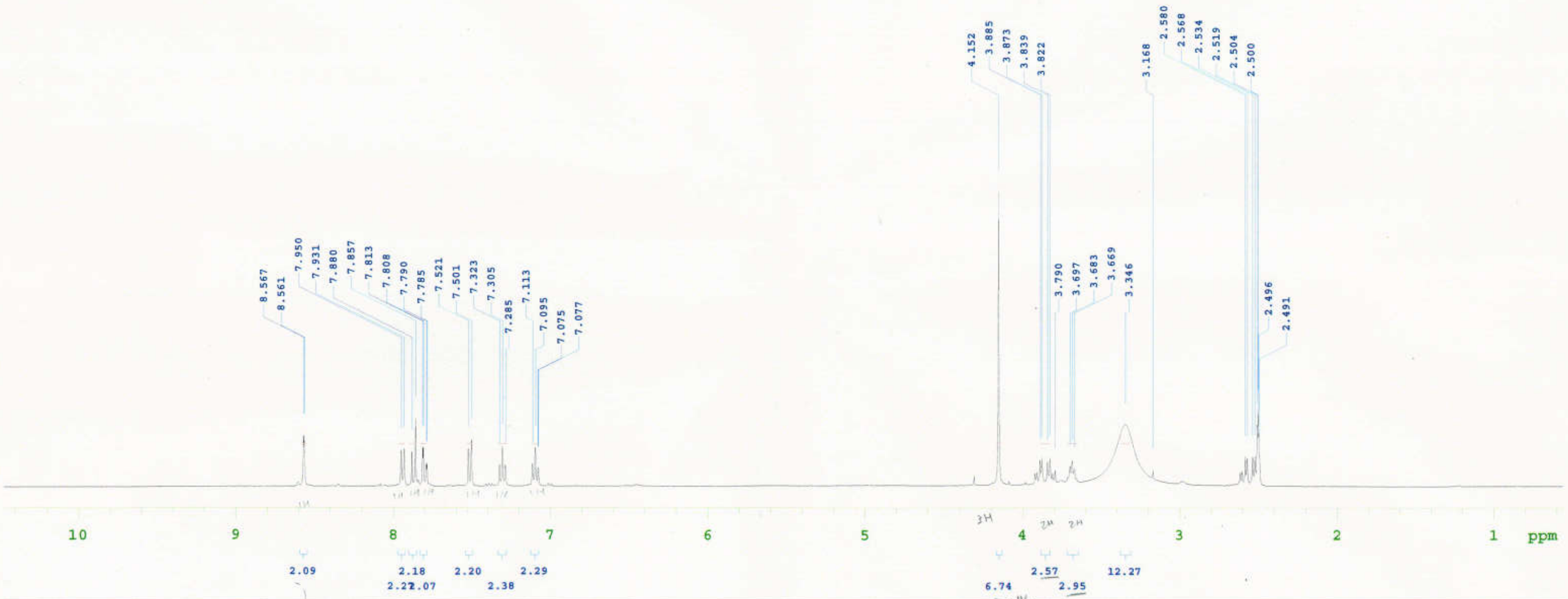

PULSE SEQUENCE
Relax. delay 1.500 sec
Pulse 45.0 degrees
Acq. time 3.500 sec
Width 6410.3 Hz
16 repetitions

OBSERVE H1, 399.9114849

DATA PROCESSING
Line broadening 0.2 Hz
FT size 65536
Total time 1 minute

2H

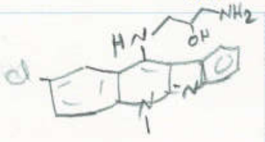

Solvent: dmso
Ambient temperature
Operator: walkup
VNMRS-400 "Varian-400MR"

sh ~~589~~ 589

File: exp

Pulse Sequence: s2pul

File: /home/walkup/vnmrsys/data/data/s2pul_001.fid

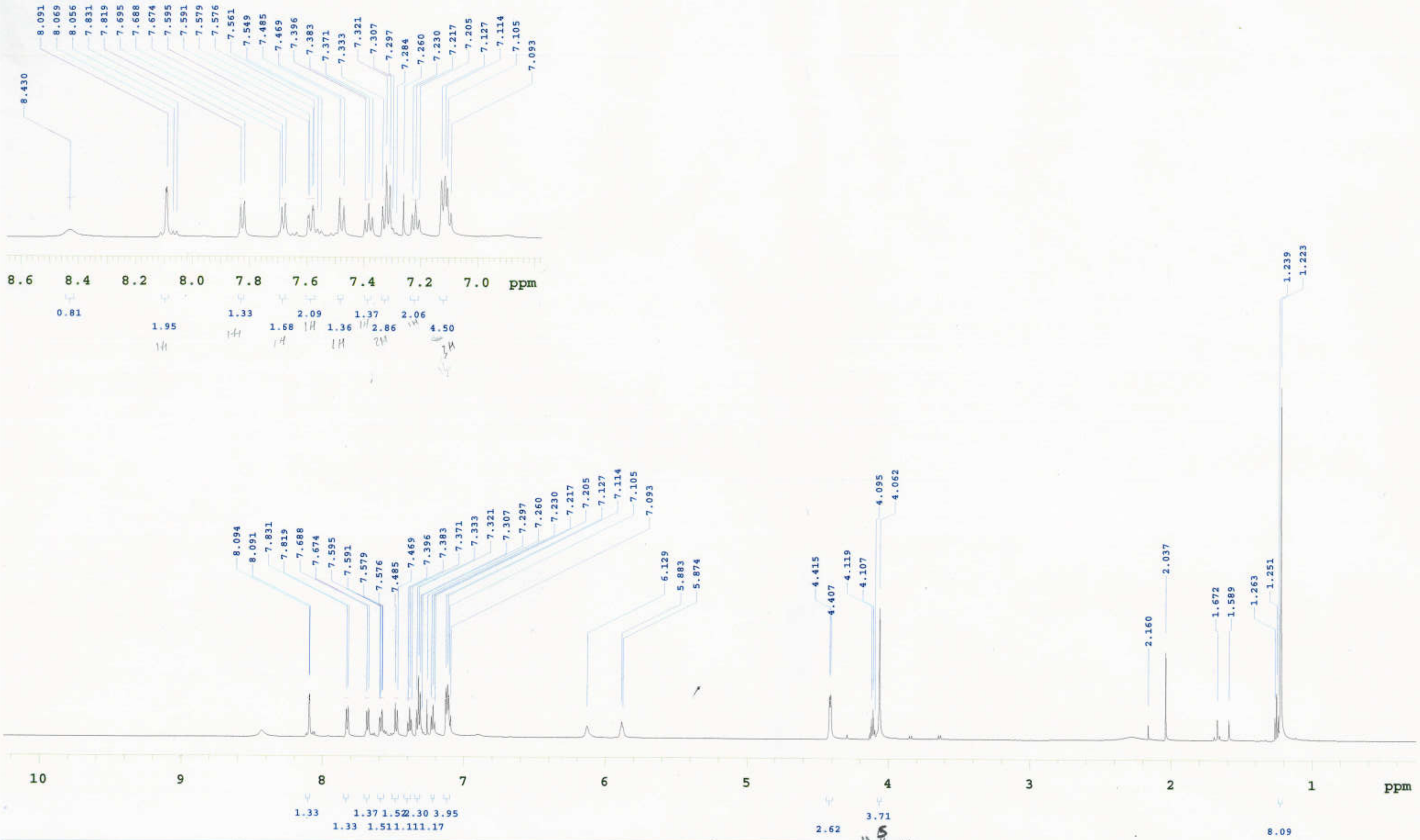

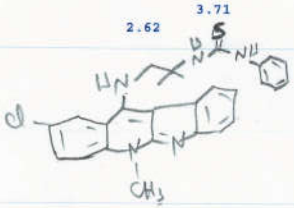

PULSE SEQUENCE
Relax. delay 1.592 sec
Pulse 45.0 degrees
Acq. time 3.408 sec
Width 9615.4 Hz
16 repetitions

OBSERVE H1, 599.7712258

DATA PROCESSING
Line broadening 0.3 Hz
FT size 65536
Total time 1 minute

Solvent: cdcl3
Ambient temperature
Operator: walkup
VNMRS-600 "varian600"

sh 574

File: Data not saved yet

Pulse Sequence: s2pul

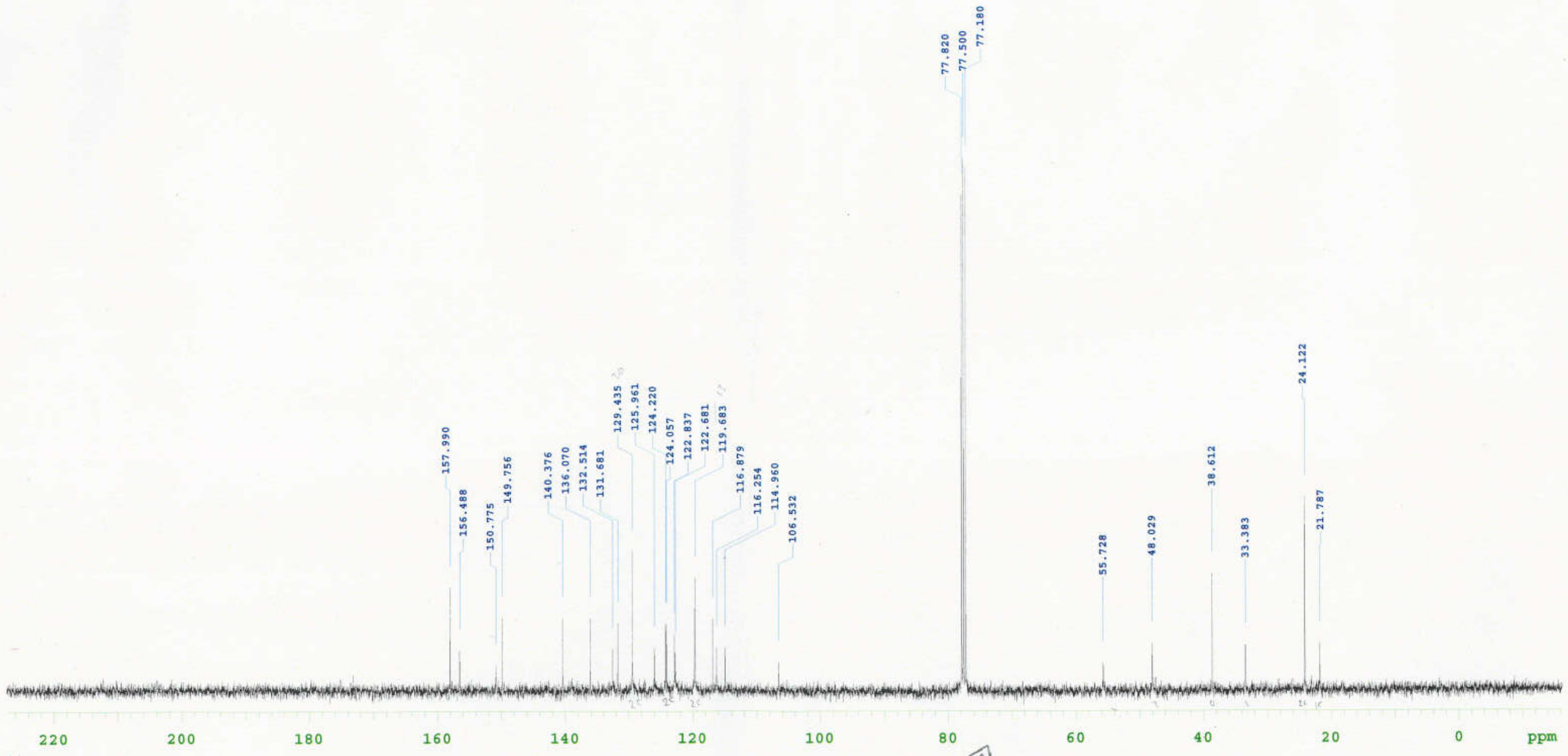

PULSE SEQUENCE<br>Relax. delay 0.700 sec<br>Pulse 45.0 degrees<br>Acq. time 1.300 sec<br>Width 24509.8 Hz<br>512 repetitions	OBSERVE C13, 100.5573007<br>DECOUPLE H1, 399.9115921<br>Power 42 dB<br>continuously on<br>WALTZ-16 modulated	DATA PROCESSING<br>Line broadening 1.0 Hz<br>FT size 65536<br>Total time 17 minutes	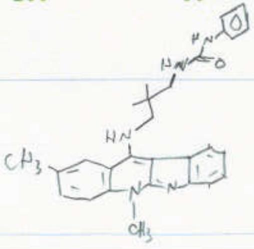	Solvent: cdcl3<br>Ambient temperature<br>Operator: walkup<br>VNMRS-400 "Varian-400MR"	sh 557 c13<br>File: exp<br>Pulse Sequence: s2pul

File: /home/walkup/vnmrsys/data/data/s2pul_001.fid

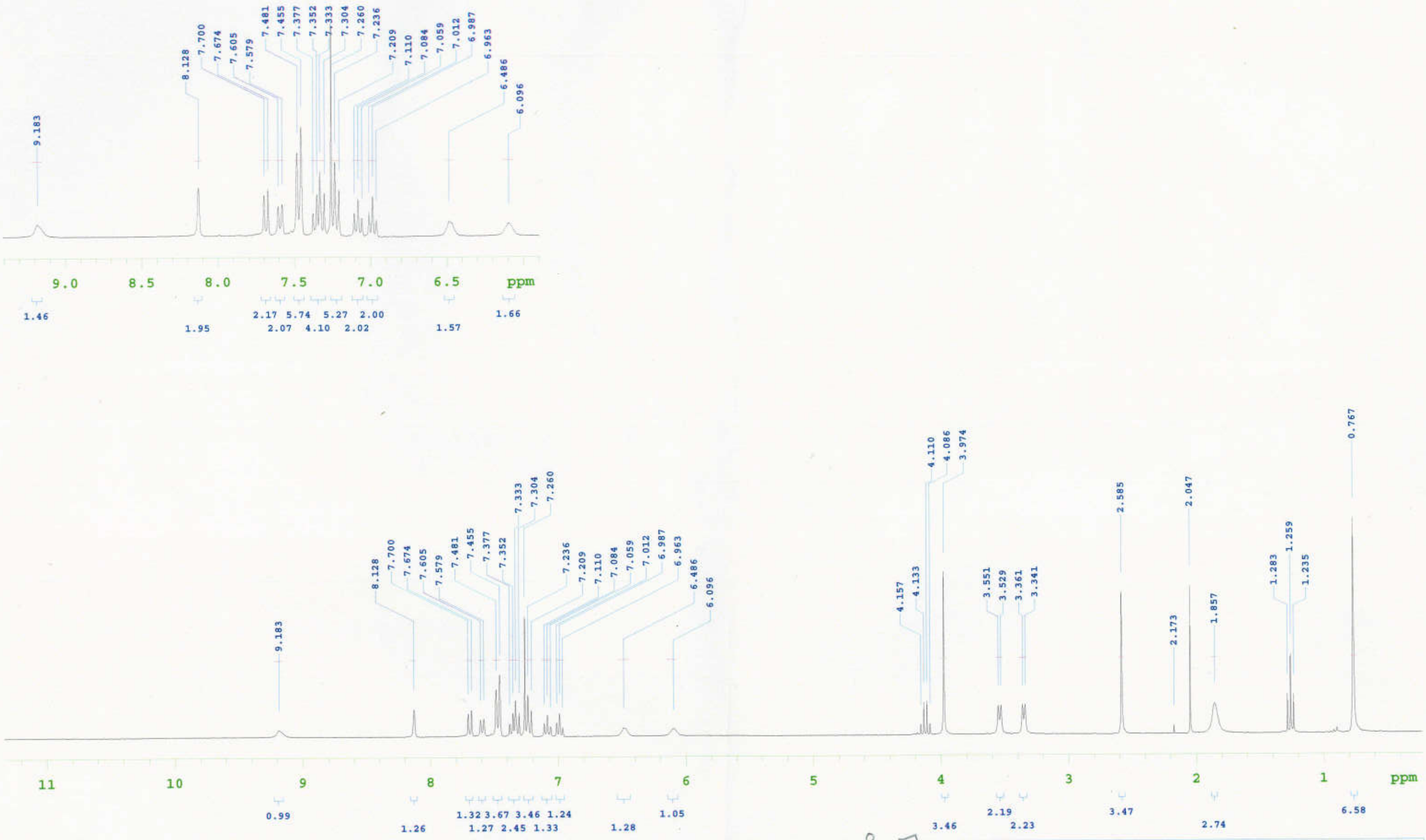

PULSE SEQUENCE
Relax. delay 1.400 sec
Pulse 45.0 degrees
Acq. time 3.597 sec
Width 4803.1 Hz
16 repetitions

OBSERVE H1, 300.0673670

DATA PROCESSING
Line broadening 0.2 Hz
FT size 65536
Total time 1 minutes

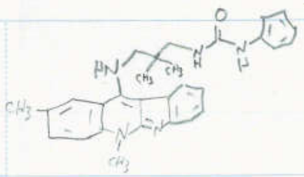

Solvent: cdcl3
Ambient temperature
Operator: walkup
Mercury-300 "Varian-NMR"

sh 557

File: exp

Pulse Sequence: s2pul

File: /home/walkup/vnmrsys/data/data/s2pul_001.fid

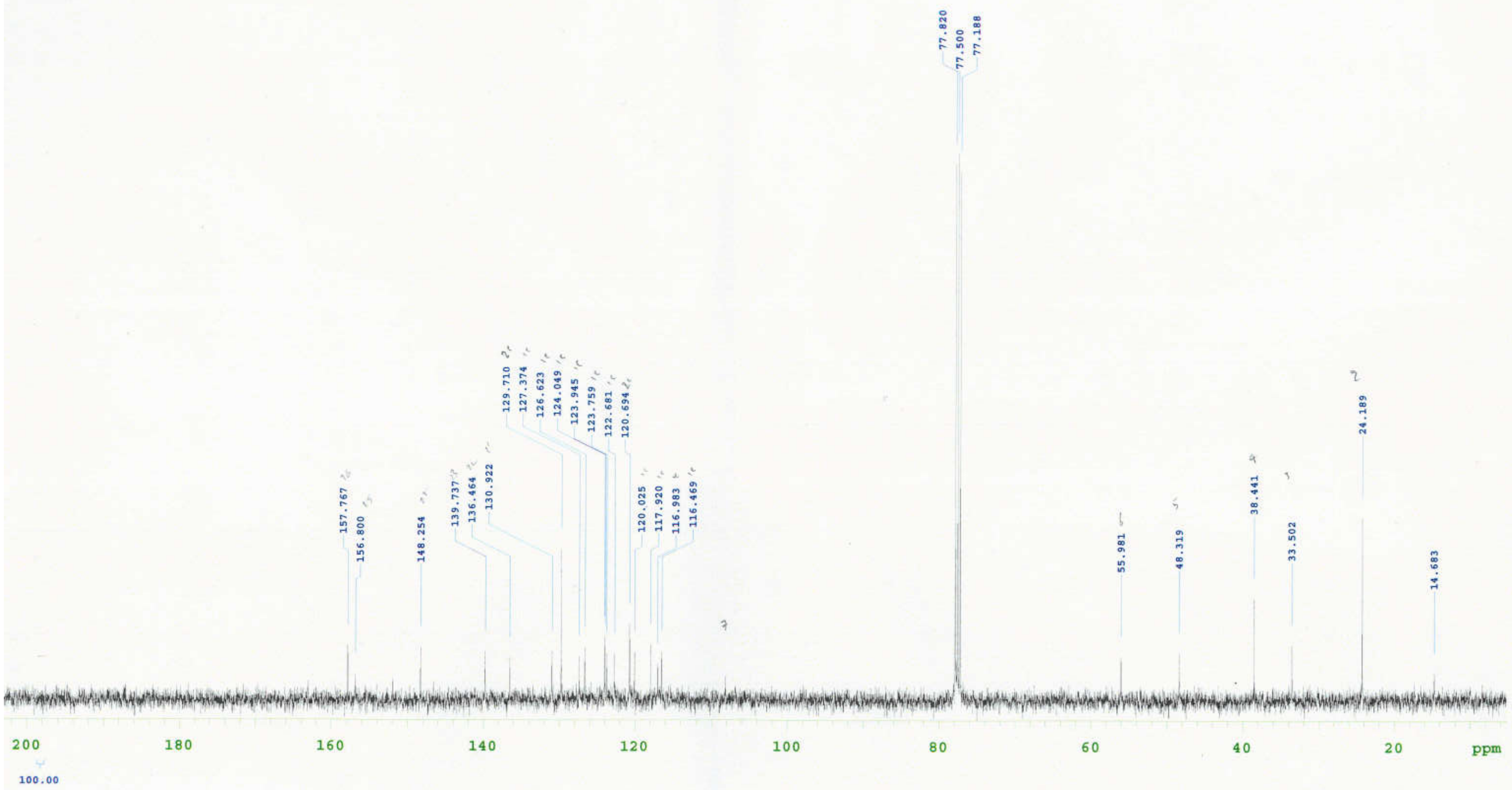

LSE SEQUENCE<br>Relax. delay 0.700 sec<br>Pulse 45.0 degrees<br>Acq. time 1.300 sec<br>Width 24509.8 Hz<br>512 repetitions	OBSERVE C13, 100.5572962<br>DECOUPLE H1, 399.9115921<br>Power 42 dB<br>continuously on<br>WALTZ-16 modulated	DATA PROCESSING<br>Line broadening 1.0 Hz<br>FT size 65536<br>Total time 17 minutes	Solvent: cdcl3<br>Ambient temperature<br>Operator: walkup<br>VNMRS-400 "Varian-400MR"	sh563c13<br>File: exp<br>Pulse Sequence: s2pul

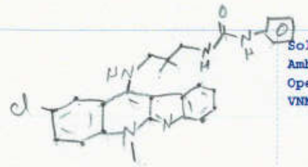

File: /home/walkup/vnmrsys/data/data/s2pul_001.fid

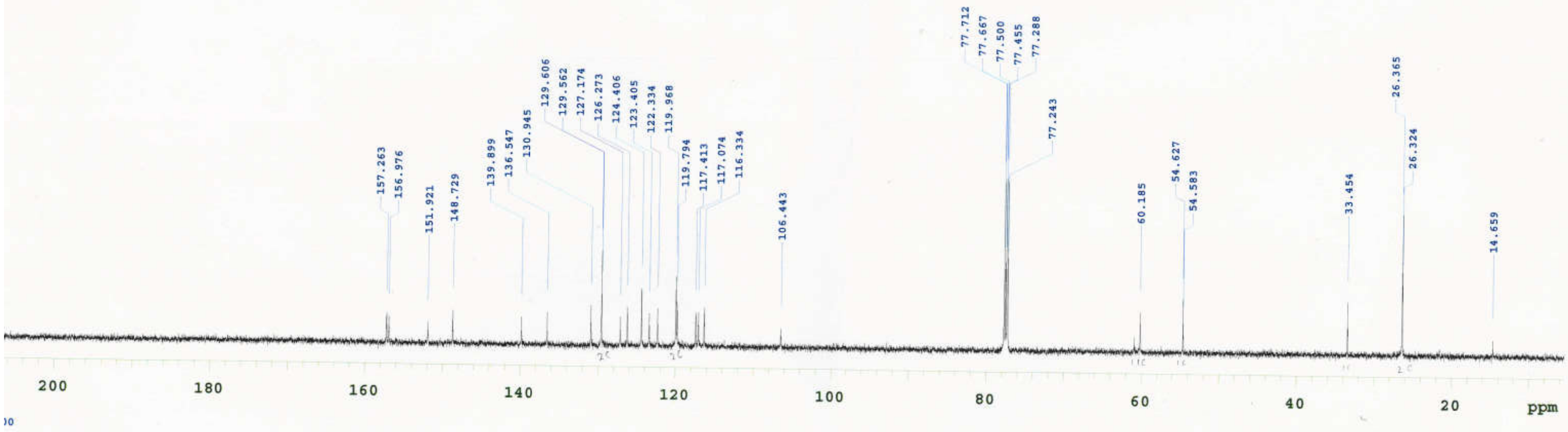

.SE SEQUENCE
:lax. delay 0.700 sec
ılse 45.0 degrees
:q. time 1.300 sec
.dth 36764.7 Hz
.2 repetitions

OBSERVE C13, 150.8125237
DECOUPLE H1, 599.7742324
Power 40 dB
continuously on
WALTZ-16 modulated

DATA PROCESSING
Line broadening 1.0 Hz
FT size 131072
Total time 17 minutes

3d

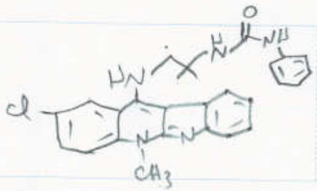

Solvent: cdcl3
Ambient temperature
Operator: walkup
VNMRS-600 "varian600"

sh 573 c13

e: Data not saved yet

Pulse Sequence: s2pul

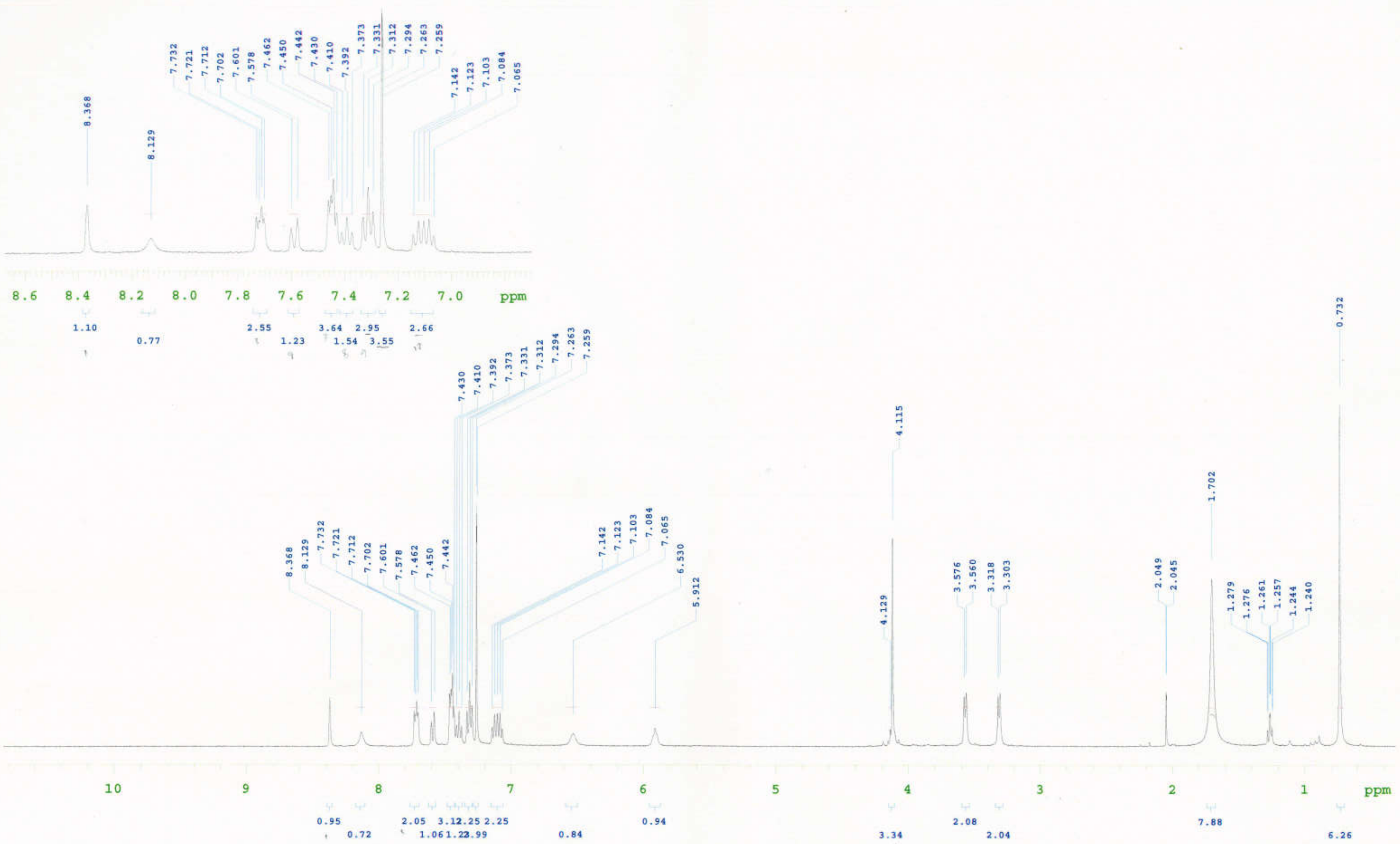

PULSE SEQUENCE
Relax. delay 1.500 sec
Pulse 45.0 degrees
Acq. time 3.500 sec
Width 6410.3 Hz
16 repetitions

OBSERVE H1, 399.9095925

DATA PROCESSING
Line broadening 0.2 Hz
FT size 65536
Total time 1 minute

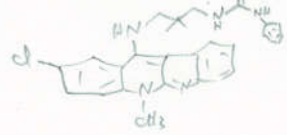

Solvent: cdcl3
Ambient temperature
Operator: walkup
VNMRS-400 "Varian-400MR"

shaban 563

File: exp

Pulse Sequence: s2pul

File: /home/walkup/vnmrsys/data/data/s2pul_001.fid

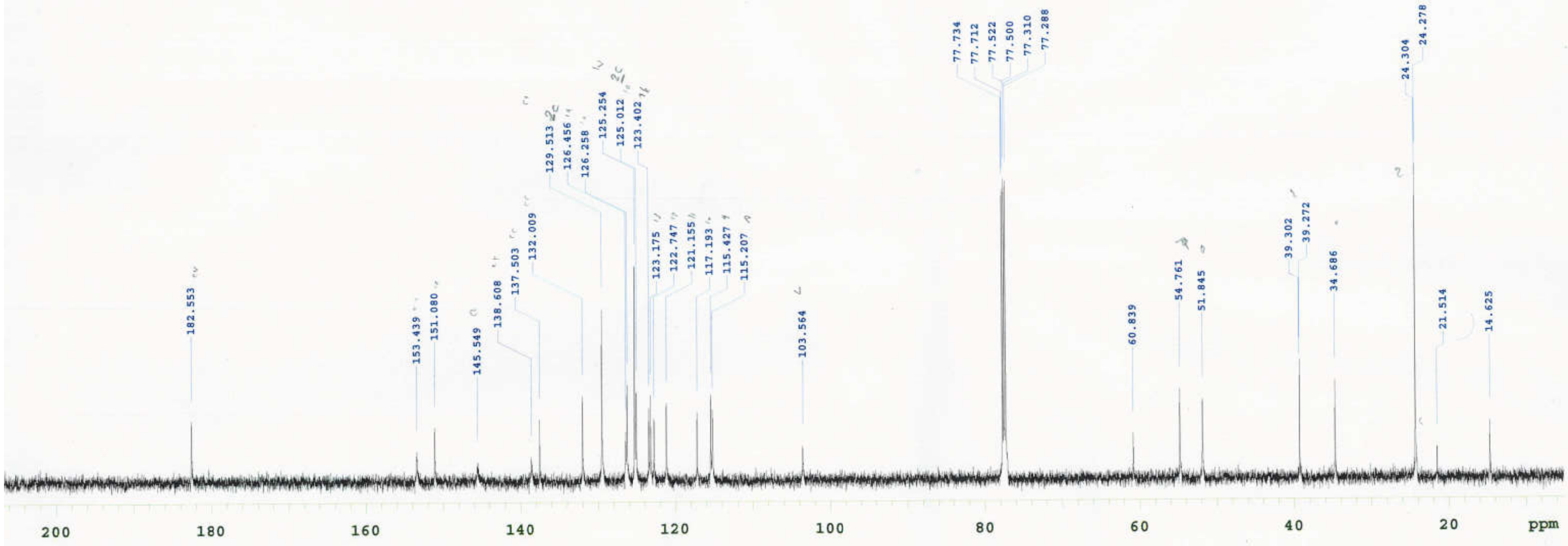

00

ULSE SEQUENCE
Relax. delay 0.700 sec
Pulse 45.0 degrees
Acq. time 1.300 sec
Width 36764.7 Hz
512 repetitions

OBSERVE C13, 150.8125282
DECOUPLE H1, 599.7742324
Power 40 dB
continuously on
WALTZ-16 modulated

DATA PROCESSING
Line broadening 1.0 Hz
FT size 131072
Total time 17 minutes

4a

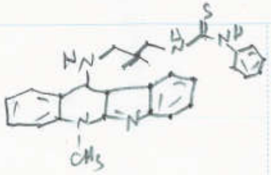

Solvent: cdcl3
Ambient temperature
Operator: walkup
VNMRS-600 "varian600"

sh 564 c13

ile: Data not saved yet

Pulse Sequence: s2pul

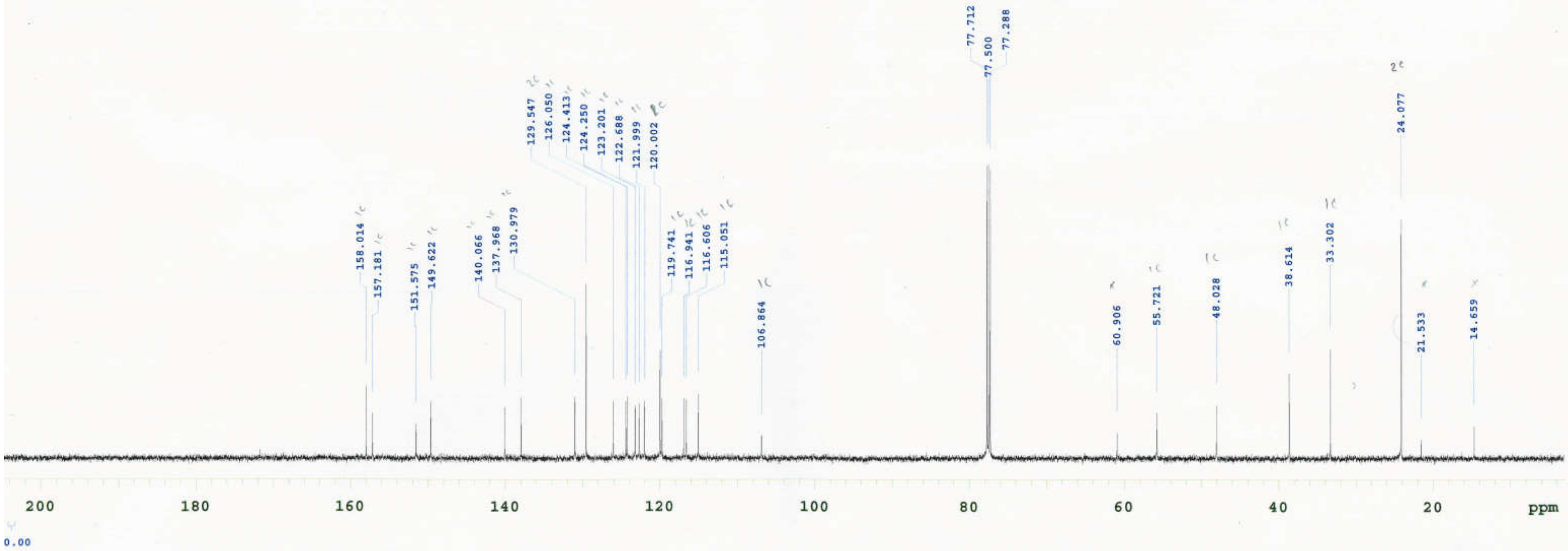

PULSE SEQUENCE
Relax. delay 0.700 sec
Pulse 45.0 degrees
Acq. time 1.300 sec
Width 36764.7 Hz
512 repetitions

OBSERVE C13, 150.8125243
DECOUPLE H1, 599.7742324
Power 40 dB
continuously on
WALTZ-16 modulated

DATA PROCESSING
Line broadening 1.0 Hz
FT size 131072
Total time 17 minutes

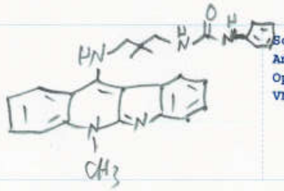

Solvent: cdcl3
Ambient temperature
Operator: walkup
VNMRS-600 "varian600"

sh 562 c13

File: Data not saved yet

Pulse Sequence: s2pul

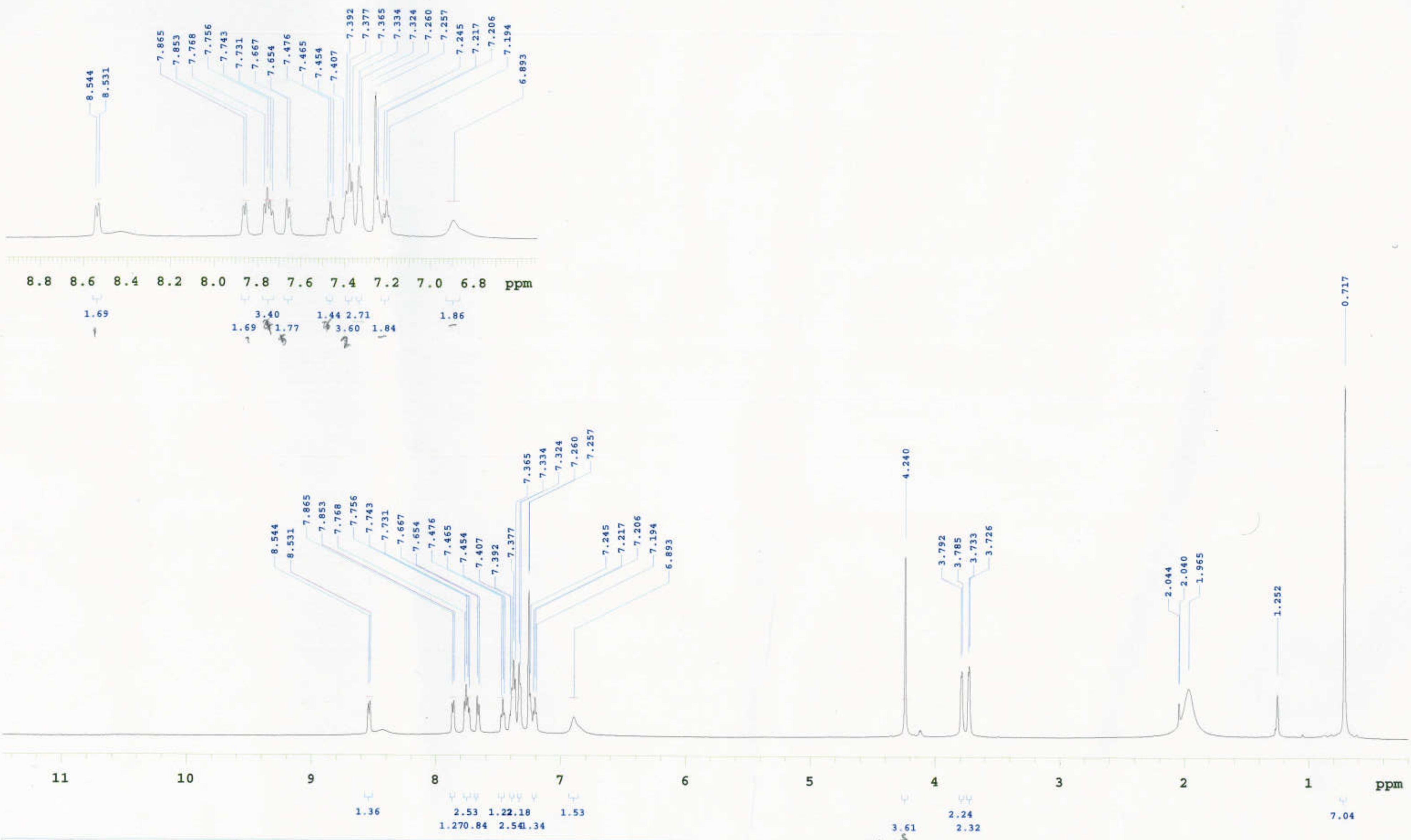

PULSE SEQUENCE
Relax. delay 1.592 sec
Pulse 45.0 degrees
Acq. time 3.408 sec
Width 9615.4 Hz
16 repetitions

OBSERVE H1, 599.7712255

DATA PROCESSING
Line broadening 0.3 Hz
FT size 65536
Total time 1 minute

4a

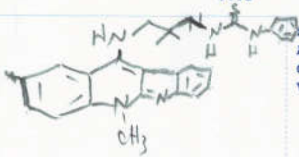

Solvent: cdcl3
Ambient temperature
Operator: walkup
VNMRS-600 "varian600"

sh 564

File: exp

Pulse Sequence: s2pul

File: Data not saved yet

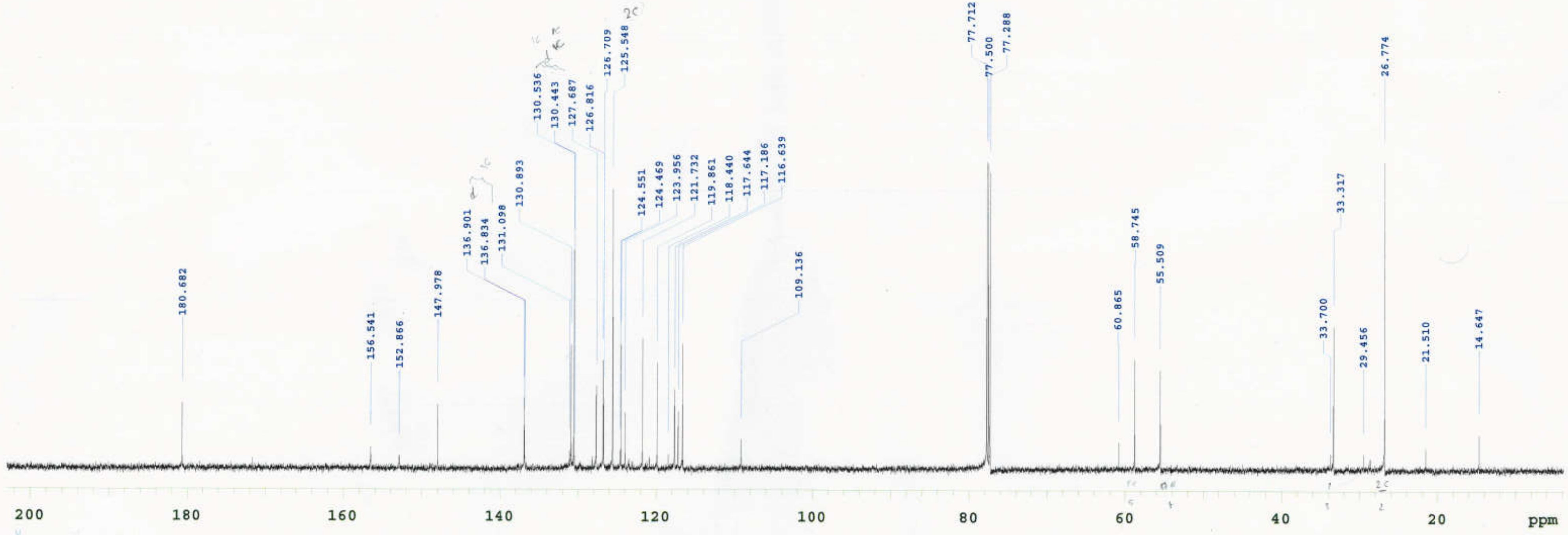

100.00

PULSE SEQUENCE<br>Relax. delay 0.700 sec<br>Pulse 45.0 degrees<br>Acq. time 1.300 sec<br>Width 36764.7 Hz<br>512 repetitions	OBSERVE C13, 150.8125260<br>DECOUPLE H1, 599.7742324<br>Power 40 dB<br>continuously on<br>WALTZ-16 modulated	DATA PROCESSING<br>Line broadening 1.0 Hz<br>FT size 131072<br>Total time 17 minutes	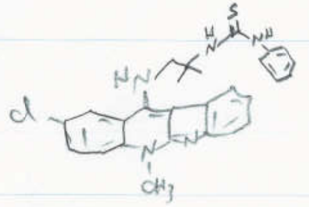	Solvent: cdcl3<br>Ambient temperature<br>Operator: walkup<br>VNMRS-600 "varian600"	sh 574 c13

File: Data not saved yet

Pulse Sequence: s2pul
